# Transition from High-Entropy to Conventional Alloys: Which Are Better?

**DOI:** 10.3390/ma14195824

**Published:** 2021-10-05

**Authors:** Emil Babić, Đuro Drobac, Ignacio Alejandro Figueroa, Mathilde Laurent-Brocq, Željko Marohnić, Vesna Mikšić Trontl, Damir Pajić, Loїc Perrière, Petar Pervan, Gyorgy Remenyi, Ramir Ristić, Amra Salčinović Fetić, Damir Starešinić, Krešo Zadro

**Affiliations:** 1 Department of Physics, Faculty of Science, University of Zagreb, 10000 Zagreb, Croatia; dpajic@phy.hr (D.P.); kzadro@phy.hr (K.Z.); 2 Institute of Physics, 10000 Zagreb, Croatia; ddrobac@ifs.hr (Đ.D.); marohnic@ifs.hr (Ž.M.); vmiksic@ifs.hr (V.M.T.); pervan@ifs.hr (P.P.); dstaresinic@ifs.hr (D.S.); 3 Institute for Materials Research—UNAM, Mexico City 04510, Mexico; iafigueroa@unam.mx; 4 ICMPE, Universite Paris Est, 94320 Thiais, France; laurent-brocq@icmpe.cnrs.fr (M.L.-B.); perriere@icmpe.cnrs.fr (L.P.); 5 Institut Neel—CNRS, 38042 Grenoble, France; gyorgy.remenyi@neel.cnrs.fr; 6 Physics Department, Josip Juraj Strossmayer University, 31000 Osijek, Croatia; ramir.ristic@fizika.unios.hr; 7 Department of Physics, Faculty of Science, University of Sarajevo, 71000 Sarajevo, Bosnia and Herzegovina; amra.s@pmf.unsa.ba

**Keywords:** compositional complex alloys, high-entropy alloys, amorphous alloys, electronic structure, atomic structure, thermal properties, hardness, magnetic properties

## Abstract

The study of the transition from high-entropy alloys (HEAs) to conventional alloys (CAs) composed of the same alloying components is apparently important, both for understanding the formation of HEAs and for proper evaluation of their potential with respect to that of the corresponding CAs. However, this transition has thus far been studied in only two types of alloy systems: crystalline alloys of iron group metals (such as the Cantor alloy and its derivatives) and both amorphous (a-) and crystalline alloys, TE-TL, of early (TE = Ti, Zr, Nb, Hf) and late (TL = Co, Ni, Cu) transition metals. Here, we briefly overview the main results for the transition from HEAs to CAs in these alloy systems and then present new results for the electronic structure (ES), studied with photoemission spectroscopy and specific heat, atomic structure, thermal, magnetic and mechanical properties of a-TE-TL and Cantor-type alloys. A change in the properties of the alloys studied on crossing from the HEA to the CA concentration range mirrors that in the ES. The compositions of the alloys having the best properties depend on the alloy system and the property selected. This emphasizes the importance of knowing the ES for the design of new compositional complex alloys with the desired properties.

## 1. Introduction

The traditional approach to the development of new materials based on lightly alloyed base elements has been progressively less successful in recent decades. The main reason behind this has been a lack of new base elements. Fortunately, a new alloy design based on multi-principal element solid solutions (not having a single base element) which can reinvigorate the discovery of new materials was introduced at the beginning of this century. First applied to amorphous alloys [1,2] in an effort to discover new bulk metallic glasses (BMG), this design soon spread to crystalline alloys (so-called high-entropy alloys, HEAs [3,4,5]) and then to intermetallic and ceramic compounds, becoming a forefront of research in materials science [6]. It is important to note that HEAs and BMGs share some common features, such as elemental disorder and, more often than not, phase metastability which strongly characterizes all their properties. HEAs were originally defined as an alloy of five or more elements with concentrations between 5 and 35 atomic % [3]. The field was later expanded to include all complex concentrated alloys (or compositional complex alloys, CCAs [7]) with as few as three principal elements and less stringent requirements on their concentrations. An obvious advantage of the new design is that, in contrast to traditional designs, it explores the middle section of the multicomponent phase diagrams which makes a virtually unlimited number of new alloys and compounds available for research and possible application [5,6,8].

The unique opportunity to greatly advance our knowledge and achieve industrial application of compositional complex alloys has aroused huge research efforts into the design, fabrication and study of HEAs. The research of HEAs alone has resulted, thus far, in several hundreds of new alloys, a very large number of research papers, tens of review papers (e.g., [9,10,11,12,13,14,15]) and books [16,17]. As a result of all these efforts, large progress has been made in the knowledge and understanding of HEAs. Several technologically relevant alloys, such as those with excellent low- and high-temperature mechanical properties as well as good oxidation and irradiation resistance, have been discovered [5,13,14]. On a more fundamental side, a new strengthening mechanism—magnetic hardening [18,19]—has been found. Further, some intriguing phenomena including the Kondo effect [20], quantum critical behavior [21] and the boson peak [22] have been observed. Moreover, several important problems in contemporary physics including the localization of electrons and phonons, various percolation phenomena at different crystal lattices, metal–insulator transitions and the nature of the boson peak in disordered systems can be studied effectively using HEAs [22,23,24,25,26].

However, research of HEAs has been mostly oriented towards the development of new structural materials and thus focused on their mechanical properties and microstructures [5,6,7,8,9,10,11,12,13,14,15,16,17,18,19,27]. Accordingly, the majority of studied HEAs are based on the iron (3d) group of elements, followed, to a lesser extent, by refractory elements. The research of the other five groups of similar elements known to form HEAs [13] has, thus far, been rather limited [6,14,15]. Therefore, the distribution of research on HEAs has been quite uneven, both as regards topics and alloy families [6,13,14,15], which affects the progress in this field [22,23,24,25,26]. The lists of some topics and HEA/CCA systems which deserve more intense study can be found in recent reviews of the literature [5,9,10,11,12,13,14,15,16,17] and our recent reports [22,23,24,25,26]. As regards new CCAs, “nonlinear alloys” built from unexpected combinations of elements (such as combinations of 3d transition metals with refractory ones [1,2,22,23,24,25,26]) have been proposed [6].

The research of their physical properties is still insufficient, despite their potential as functional materials [5,28], and the conceptual importance of these properties [20,21,22,23,24,25,26,29]. The main problem in the selection of HEA/CCA systems possessing desirable properties is the limited conceptual understanding of these complex multicomponent systems. The main hindrance for such an understanding of both crystalline (c-) and amorphous (a-) HEAs and CCAs is probably the lack of detailed insight into their electronic structures (ESs), which, in metallic systems, determine all intrinsic properties [5,15,17,18,19,20,21,22,23,24,25,26,30,31,32,33,34,35,36,37,38,39,40,41,42,43,44] including the mechanical ones [5,15,18,19,31]. Fortunately, the number of theoretical studies of ESs and selected properties of CCAs has increased dramatically over the last ten years and, at present, largely exceeds the number of experimental studies of their ES. As noted in our previous reports [22,23,24], a combination of experimental results from photoemission spectroscopy (PES) and low-temperature specific heat (LTSH) with theoretical calculations is required to obtain a reliable and quantitative insight into the ES of the studied system. At present, there is a very small number of PES studies that have been performed on HEAs/CCAs [22,23,24,25,26].

**Figure 1:**
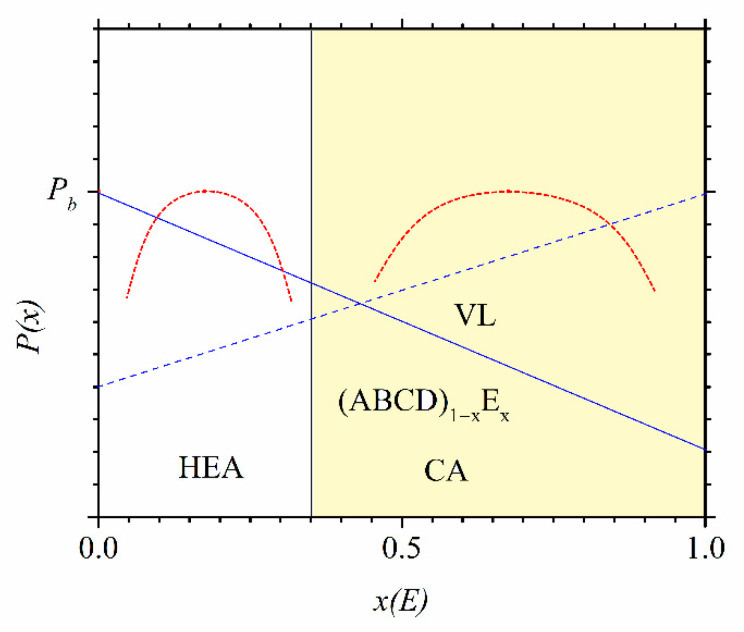
Some variations of a property *P* of a hypothetical (ABCD)_1−x_E_x_ alloy with *x*. Straight lines illustrate variation according to Vegard’s law [60]. *P*_b_ denotes a maximum value of *P*.

Two important issues in CCAs have been relatively poorly studied thus far. One is the transition from HEAs/CCAs to conventional alloys (CAs) based on one or a maximum of two principal elements with the same chemical make-up [24,25,26,37,45,46,47,48,49,50,51,52]. The other is the disentanglement of the effects of topological and chemical disorder on their properties [23,24,51,53,54,55,56,57,58]. The study of the transition from HEAs/CCAs to CAs is apparently important, both for understanding the formation and stability of HEAs/CCAs [24,25,37,38,39] and for proper evaluation of their potential with respect to that of the corresponding CAs. Thus, such a study can demonstrate whether, for a given alloy system and selected property, HEAs/CCAs outperform CAs, or not. This is illustrated in Figure 1, where some possible variations of a selected intrinsic property *P* with the concentration (*x*) of the element E in a hypothetical quinary alloy (ABCD)_1−x_E_x_ are shown. The alloy can be either amorphous or crystalline. For simplicity, we will assume that it can be prepared as a single-phase solid solution (thereafter, SS) over an extended range of *x*, covering both the HEA/CCA and CA (*x* ≥ 0.35) concentration ranges. We have extended the HEA range in Figure 1 and thereafter as well to below *x* = 0.05 [3], which is in accordance with more recent definitions of HEAs [6,13,14,15]. Since the variations of intrinsic properties with *x* in an alloy system reflect the evolution of the corresponding ES, it is clear that *P* can accept the most desirable value (*P_b_*) at an arbitrary *x* which is not limited to the HEA concentration range. Further, when the rule of mixtures applies to a given alloy and property, *P* may vary linearly with *x*. We note that in this case, which is often assumed to apply to HEAs/CCAs, neither the largest nor the smallest value of *P* can occur in the HEA concentration range. Thus, when the rule of mixtures applies, the HEA concentration range is rather uninteresting. Moreover, the study of the thermal parameters and mechanical properties of the Cantor alloy [4], CrMnFeCoNi, and of quaternary, ternary and binary equiatomic alloys composed of its constituent elements [7] has shown that these properties depend more on the types of alloying elements than on their number. All variations of P illustrated in Figure 1 have been observed in studies of the transition from HEAs to CAs [24,25,26,45,46,47,48,51,52]. In the rest of this paper, we will discuss only the transition from HEAs/CCAs to CAs in two dissimilar alloy systems: (i) the “nonlinear alloys” composed of early (TE) and late (TL) transition metals [24,25,26,37,51,52] and (ii) these composed of 3d transition metals [45,46,47,48,49,50]. We note, however, that the alloys composed of TE and TL are advantageous in studying both the disentanglement of the effects of topological and chemical disorder and the transition from HEAs to CAs since they possess a relatively simple split band ES, strong interatomic bonding and large atomic size mismatch. All of them favor elemental and topological ordering under suitable conditions [51,53,54]. In addition, they can be prepared in an amorphous phase [59] over a broad composition range (which facilitates the study of the transition from an HEA to a CA in the same alloy system [24,25,26,52]).

The first step towards the research of the transition from HEAs to CAs was established by the group of Yeh [49]. Although their research of Al_x_CrFeCoNi alloys remained within the HEA concentration range, it showed the large potential of compositional tuning of the atomic structure and properties of HEAs. Moreover, this study was a basis for the present-day predictions of the crystalline structures of 3d transition metal HEAs and their derivatives which are based on their average number of valence electrons per atom, VEC [61]. As described in some detail elsewhere [45], subsequent studies of non-equiatomic 3d CCAs were mainly focused on tuning their properties by using relatively small changes in their composition (which was deemed necessary to remain within the same SS phase). Therefore, it came as a surprise when a group at ICMPE in Thiais discovered [45] that under suitable conditions, (CrMnFeCo)_1−x_Ni_x_ alloys can be prepared in a face-centered cubic (FCC) phase over a broad composition range, extending from the Cantor HEA [4] (*x* = 0.2) to pure Ni (*x* = 1).

Strong interatomic interactions in “nonlinear” TE_1−x_TL_x_ CCAs make it even more difficult to remain within the same phase for any larger change in composition. Accordingly, with one exception [51], all studies of crystalline TE-TL CCAs investigate a rather narrow composition range, mostly centered around *x* = 0.5 [28,29,54], which is suitable for both shape memory alloys [28] and very strong and stable alloys possessing an ordered B2 phase [51,54]. However, it was noted [22] that the same alloys can be prepared in an amorphous state over a broad composition range covering both HEAs and CAs. This is beneficial for the study of the transition from HEAs to CAs and the disentanglement of the effects of compositional and topological disorder and may also enable a better understanding of a-HEAs/CCAs. Accordingly, a broad collaboration coordinated from Zagreb was set to study these issues [22,23,24,25,26,52,62].

Here, we compare the effect of varying the composition over a broad range covering both HEA and CA concentrations on several properties of selected Cantor types of 3d transition metal alloys [45,46,47,48], e.g., (CrMnFeCo)_1−x_Ni_x_, with that in three quinary amorphous TE-TL alloy systems [22,23,24,25,26,37,52], e.g., (TiZrNbCu)_1−x_Ni_x_. Only those alloy systems which could be prepared in a single phase up to *x* ≥ 0.43 (thus entering into the CA concentration range) will be discussed. First, we compare the thermophysical parameters of these two alloy families. These parameters are commonly used in empirical criteria for phase selection in HEAs [12] and correctly predict formations of very different phases in our 3d transition metal and TE-TL alloy families. Next, we compare phase diagrams and associated thermal parameters of selected alloys. We also compare the measured thermal parameters with those calculated by using the rule of mixtures. For TE-TL alloys, we briefly discuss the dichotomy between the rather good thermal stability of a-HEAs and their very moderate glass-forming ability, GFA [52,63]. We also provide, to our knowledge, the first results for the variation of crystallization enthalpies with the composition across the transition from HEAs to CAs. In discussing the atomic structures, we first show that the primary crystallization product in the studied amorphous TE-TL alloys is a metastable body-centered cubic (BCC) phase. Assuming local BCC-like atomic arrangements in the amorphous phase of these alloys, we calculate the corresponding average lattice parameters and compare their variations with those observed in 3d transition metal alloys (possessing an FCC crystal structure). In both alloy families, depending on the chosen principal component, the variations of lattice parameters with the composition can either follow Vegard’s law or show strong deviations from this law (e.g., [45]). The difference between the two alloy families shows up the best in their ESs. The first study of the variations of the electronic density of states (DOS) within the valence band in 3d transition metal CCAs, performed with PES, showed a smooth variation of DOS with energy, which is very different from the split band shape of DOS in the TE-TL alloy family [22,23,24,25,26]. In both alloy families, the magnetic and mechanical properties are related to the corresponding ESs, correlations and atomic structures. This relationship is particularly simple in amorphous TE-TL alloys due to their relatively simple ES and the absence of long-range magnetic order. The main message from our study is that some properties of very different alloy systems can show qualitatively similar behaviors. The results for the studied alloys are compared with a few existing results for similar multicomponent alloys (e.g., [50,51]). The results for our quinary amorphous alloys are also compared with those for corresponding binary and ternary TE-TL amorphous alloys.

## 2. Materials and Methods

The materials, methods of preparation and characterization of the samples and some techniques of performing measurements employed for the Cantor-type 3d transition metal alloys and quinary TE-TL metallic glasses (MG) have been described in some detail elsewhere [22,23,24,25,26,45,46,47,48,52]. For completeness, we briefly describe these already known issues and use somewhat more space for new ones. All the studied alloys have the composition (ABCD)_1−x_E_x_ (e.g., Figure 1), where A, B, C and D are the elements in equiatomic proportion, E is the element whose content is made to vary, and *x* is the atomic fraction.

### 2.1. Cantor Type of Alloys

The ingots of ten (CrMnFeCo)_1−x_Ni_x_ (0.2 ≤ *x* ≤ 0.92) and five (CrMnCoNi)_1−x_Fe_x_ (0 ≤ *x* ≤ 0.5) alloys were prepared at ICMPE from high-purity components (≥99.95%) by using high-frequency electromagnetic induction melting in a water-cooled copper crucible under a pure He atmosphere, followed by suction casting into the shape of cylinders with 3 mm in diameter [45]. (The same procedure was used for the production of ingots of three other alloy systems in which the E = Cr, Mn or Co content (*x*) was varied [47,48].) Then, 2–3 mm-thick slices of ingots wrapped in tantalum foils were annealed at 1373 K for 6 h under a pure He atmosphere [45]. The annealed samples, still in a He atmosphere, were quickly cooled down to room temperature. The plate-like samples required for magnetization and PES measurements were, after casting, cold rolled down to the thickness of 0.5 mm (83% of reduction in thickness) and then annealed at 1373 K for 6 h under a pure He atmosphere. All subsequent characterizations and measurements were performed on annealed (homogenized) samples. Samples for microstructural and mechanical investigations were prepared by mechanical grinding using 1200 to 4000 grit abrasive papers, followed by a final polishing step using a vibratory table and a 0.04 μm colloidal silica [47].

After the homogenization annealing, X-ray diffraction (XRD) was performed in a PANalytical X’Pert Pro (PANalytical, Palaiseau, France) diffractometer using Co-Kα radiation at a wavelength of 0.178897 nm and an angular step of 0.016°. X-ray diffractograms were refined by the Rietveld method (FullProf SUITE software) (Version Juillet 2017, Grenoble, France) and used to determine the lattice parameters [47]. XRDs of all studied alloys exhibited similar patterns, i.e., four peaks corresponding to a single FCC phase. The samples were also characterized using a Merlin Field Emission Gun Scanning Electron Microscope (FEG-SEM) (Zeiss, Oberkochen, Germany) coupled with an Energy Dispersive X-ray Spectrometer (EDS) from Oxford Instruments (Abingdon, UK). Two elemental mappings with a size of 1100 × 800 μm^2^ and one mapping with a size 110 × 80 μm^2^ were performed to measure the average chemical composition and to ensure chemical spatial homogeneity. In addition, on some samples (e.g., those with *x* = 0.5 and 0.6 Ni), the local composition and homogeneity were measured at 30 spots with EPMA using the Cameca SX100 device (CAMECA, Gennevilliers, France) and a DSC 404 F1 Pegasus calorimeter (Netzsch, Selb, Germany) was used to perform differential scanning calorimetry on all (CrMnFeCo)_1−x_Ni_x_ samples. About 80 mg of each sample in an alumina crucible was heated up to 1773 K at a rate of 20 K/min under a pure Ar flow and then cooled down to room temperature at the same rate. After several cycles of ramping the temperature of the same sample from room temperature up to 1773 K, its melting, *T*_m_, and liquidus, *T*_l_, temperatures became reproducible to within 1 K.

Nanoindentation was performed on polished samples with a TI 950 indenter (Hysitron-Brucker, Thiais, France) which was equipped with a Berkovich tip. Scanning probe microscopy (Hysitron-Brucker, Palaiseau, France), which consists in scanning the sample surface with the nanoindenter tip, was used to obtain 10 × 10 μm^2^ images of the surface topography. Values between 2 and 5 nm and 0.3 and 1.5° were determined for roughness and tilt, respectively. Thus, performed indents with maximum depths from 300 to 400 nm were not influenced by the surface quality. For each indentation, the loading and unloading rates were 1 mN s^−1^, and the maximum load (up to 12 mN, depending on the sample) was maintained for 5 s. At least 20 indentations separated by 20 μm were performed on each sample. The standard deviation of the calculated nanohardness, *H*_nano_, was around 5%. A detailed description of hardness measurements can be found in Reference [47].

The measurements of magnetic susceptibilities were performed at the Institute of Physics in Zagreb. The samples with transition temperatures below about 380 K were measured with a specially designed high-sensitivity ac susceptometer [64,65] suitable for measurements in the temperature range 1.5–400 K. The main design concept is that magnetic coils (primary, secondaries) are immersed in a cryogenic liquid (LHe, LN2), while the sample holder, made of a sapphire rod (partly flattened for placing the sample), is inserted inside a coaxial high-vacuum chamber, and the temperature is regulated by the use of an appropriate wire wound heater. A constant temperature of coils grants easy compensation of all disbalance voltages and a fixed phase relationship between the applied and induced AC voltages, and thus the signal stability in the course of measurement. This device operates in the frequency range 1–1000 Hz and can also measure the magnetic hysteresis loops using AC magnetic fields up to 1 kOe. The transition temperature and the corresponding critical exponent were measured in a compensated Earth magnetic field, using a low excitation field with a typical amplitude of *H*_0_ = 0.1 Oe, applied along the length of the sample with dimensions 10 × 2 × 0.5 mm^3^. The samples with Curie temperatures above 380 K were investigated in another device at the Institute of Physics in Zagreb designed for studies of the magnetic after effect (MAE) in the temperature range from room temperature up to 1100 K. In this high-temperature system, the coils are immersed in low-viscosity oil and air cooled by forced circulation. The sample holder itself represents, in fact, the “oven”. A platinum wire wound heater was used for heating the central rod-like ceramic (boron nitride, alumina, etc.) with a gap for the sample. Several tiny wall ceramic tubes and molybdenum radiation shields were added coaxially. The maximal required heating power to cover the whole temperature range was 12 W. This device can also be used for standard ac susceptibility measurements within the same range of amplitudes and frequencies of exciting magnetic fields and the same alignment of the field as that employed in the low-temperature device [64]. Both devices use calibrated thermocouples for temperature measurements and possess accurate temperature controllers.

Since the same experimental setup was used for the investigations of magnetization and photoemission spectra in both the Cantor types of alloys and quinary TE-TL MGs, they will be described in the next section devoted to MGs.

### 2.2. Quinary TE-TL Metallic Glasses

The ingots of: seven (TiZrNbCu)_1−x_Co_x_ alloys (*x* = 0, 0.1, 0.2, 0.25, 0.32, 0.43 and 0.5) [52], eight (TiZrNbCu)_1−x_Ni_x_ alloys (*x* = 0, 0.125, 0.15, 0.2, 0.25, 0.35, 0.43 and 0.5) [24,37] and eleven (TiZrNbNi)_1−x_Cu_x_ alloys (*x* = 0, 0.05, 0.12, 0.15, 0.2, 0.25, 0.32, 0.35, 0.43, 0.5 and 0.52) [25] were prepared from high-purity components (≥99.8 at %) by arc melting in high-purity argon in the presence of a titanium getter. All ingots were flipped and remelted five times to ensure complete melting and good mixing of components. Samples in the form of ribbons with a thickness of about 25 μm of each alloy were fabricated by melt spinning molten alloy on the surface of a copper roller rotating at the speed of 25 m/s in a pure helium atmosphere. Casting with controlled parameters resulted in ribbons with similar cross-sections and surface appearances, and thus with the amorphous phases having a similar degree of quenched disorder.

All as-cast ribbons were studied by X-ray diffraction (XRD) using a Bruker Advance powder diffractometer (Bruker Corporation, Billerica, MA, USA) with a Cu-Kα source [23,24,25,37]. The XRD patterns showed that all samples, except for those with *x* = 0 and samples with *x* = 0.5 Co and *x* = 0.52 Cu, were X-ray amorphous. XRD was also used to study the crystallization products in the sample with *x* = 0.125 Ni annealed at different temperatures. The atomic structure of amorphous samples was also investigated using synchrotron-based high-energy X-ray diffraction (HEXRD) [24,25,62] at the Diamond Light Source, Didcot, UK. A piece of sample ribbon was illuminated with a monochromatic beam of 0.01545 nm wavelength for a total time of 240 s. After every sample measurement, the air scattering signal was measured under the same experimental conditions. All HEXRD experiments were carried out in transmission mode using a flat-panel Pixium RF4343 detector (Thales Group, Paris, France) [24,25].

The ribbons which appeared in X-ray amorphous form were further studied by differential scanning calorimetry (DSC) and thermogravimetric analysis (TGA) using a Thermal Analysis DSC-TGA instrument (TA Instruments, New Castle, DE, USA). Thermal measurements were performed up to 1600 K with a ramp rate of 20 K/min. The values of thermal parameters, including the crystallization enthalpies, were determined by using TA “Advantage” software (TA Instruments, New Castle, DE, USA). Regular calibration of DSC-TGA equipment [24,25,37] keeps the uncertainty in measured temperatures within ± 5 K. Fully amorphous as-cast ribbons were also investigated with scanning electron microscopy (SEM) using a JEOL ISM7600F microscope (JEOL Inc., Tokyo, Japan) with energy-dispersive spectroscopy (EDS) capability to determine their actual compositions and chemical homogeneity [23,24,25,52]. Elemental mapping was performed on three different areas of each sample.

The valence band structure of all as-cast amorphous alloys, as well as that of Cantor-type 3d transition metal alloys described in the previous Section 2.1., was studied by ultraviolet photoemission spectroscopy (UPS), equipped with a Scienta SES100 hemispherical electron analyzer (ScientaOmicron, Uppsala, Sweden) attached to an ultra-high vacuum chamber with a base pressure below 10^−9^ mbar [22,23,24,25,26,52] at the Institute of Physics in Zagreb. An unpolarized photon beam of energy 21.2 eV was obtained by a He^−^ discharge ultraviolet photon source (He-I). The overall energy resolution in UPS experiments was about 25 meV. The samples were cleaned by several cycles of sputtering with 2 keV Ar^+^ ions at room temperature to remove the oxygen and other contaminants from the surface.

The as-cast ribbons were also used for measurements of magnetization, magnetic susceptibility and LTSH [23,24,26,52]. The magnetization and magnetic susceptibility of all alloys, including the Cantor-type alloys from the previous section, were measured with a Quantum Design magnetometer, MPMS5, in a magnetic field *B* up to 5.5 T and temperature range of 2–350 K [24,25,32,33,37]. Since the magnetic susceptibility of all TE-TL alloy samples, except for that with *x* = 0.5 Co, showed a weak dependence on the temperature within the explored temperature range (as is usual in nonmagnetic alloys of TE and TL metals, e.g., [24,25,32,33,66,67]), in the following analysis, we will use the room temperature values. For the Cantor-type alloys, both the field dependence of magnetization at different temperatures and the temperature dependence of magnetization under field cooling and zero-field cooling conditions were studied. The measurements of LTSH on (TiZrNbNi)_1−x_Cu_x_ alloys and two (TiZrNbCu)_1−x_Co_x_ samples with *x* = 0.2 and 0.43 were performed in the temperature range 1.8–300 K using a Physical Property Measurement System (PPMS) Model 6000 from Quantum Design (San Diego, CA, USA), as previously described [23,24,26,37,68].

The microhardness of all as-cast amorphous samples was measured at room temperature. These measurements were performed using a DHV-1000Z Micro Vickers Hardness Tester device (Sino Age Development Technology, Beijing, China) equipped with pyramidal indenter with a square base, having an angle of 1370. Ten indentations were made on both sides of each sample. The loading time was 15 s, and the load was 0.981 N. The standard deviations were about 5% of the mean values. Young’s modulus, *E*, calculated from the relationship *E* = *Dv*^2^, where *v* is the velocity of ultrasonic waves along the 100 mm-long ribbon, was measured both on as-cast ribbons and the same ribbons annealed for a short time close to the glass transition temperature of a given alloy, and *D* is the corresponding mass density [23,37,66]. *E* was measured on several (TiZrNbCu)_1−x_Ni_x_ and (TiZrNbNi)_1−x_Cu_x_ alloys, and its standard deviation was around 2%.

## 3. Results and Discussion

### 3.1. Thermophysical Parameters and Elemental Distribution

The vast number of HEAs that can be designed from stable elements [10,13,69] makes searching for technologically interesting compositions by trial and error clearly inadequate [8], despite the rapid evolution of various combinatorial high-throughput methods for simultaneous production of a range of compositions (e.g., [70]). Accordingly, a large effort has been devoted to the prediction of the phase formation in HEAs [9,10,11,12,13,14,15,16,17,57,71]. Several semi-empirical criteria for the formation of different phases, single-phase solid solutions (SSs) and intermetallic compounds (IM) and their mixtures with an SS and/or an amorphous phase, a-HEAs, have been developed [9,10,11,12,13,14,15,16,17,57]. These criteria are mainly based on thermophysical parameters such as the mixing or formation enthalpy [70], ∆*H*_mix_, the ideal configurational entropy, ∆*S*_conf_, the atomic size mismatch, *δ*, the valence electron concentration, VEC, and electronegativity (see, e.g., [10,11,12,13,61]). *δ* and ∆*H*_mix_ are defined as

(1) $$\delta =\sqrt{\sum_{i=1}^{n}c_{i}\;{\left(1-\frac{r_{i}}{\sum_{j=1}^{n}c_{j}\;r_{j}}\right)}^{2}}$$

(2) $$\Delta H_{\mathrm{mix}}=\sum_{i=1,i\neq j}^{n}4\;\Delta H_{ij}^{\mathrm{mix}}\;c_{i}\;c_{j}$$

where *c*_i_ and *r*_i_ denote the atomic fraction and atomic radius of the *i*th element, respectively, and $\Delta H_{ij}^{\mathrm{mix}}$ denotes the enthalpy of mixing of the binary liquid between the *i*th and *j*th elements at an equiatomic composition [10,12].

**Figure 2:**
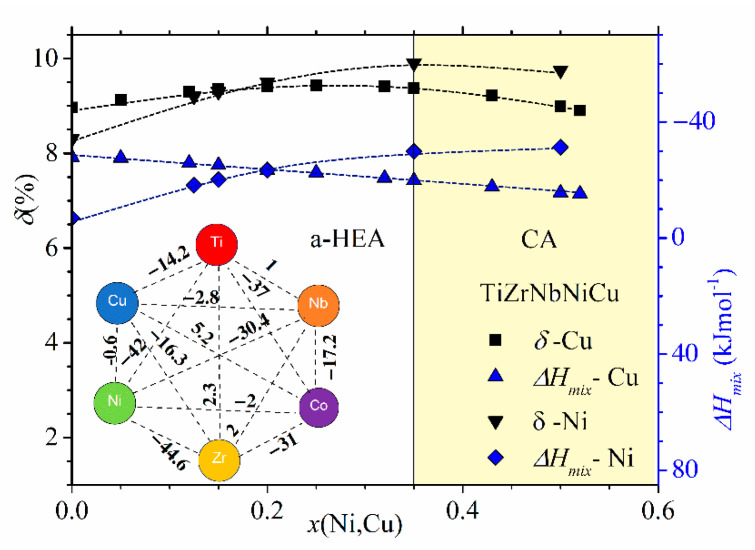
Atomic size mismatch *δ* and mixing enthalpy ∆*H*_mix_ of (TiZrNbCu)_1−x_Ni_x_ and (TiZrNbNi)_1−x_Cu_x_ alloys vs. *x*. The inset: ∆*H_mix_* between the constituent elements.

**Figure 3:**
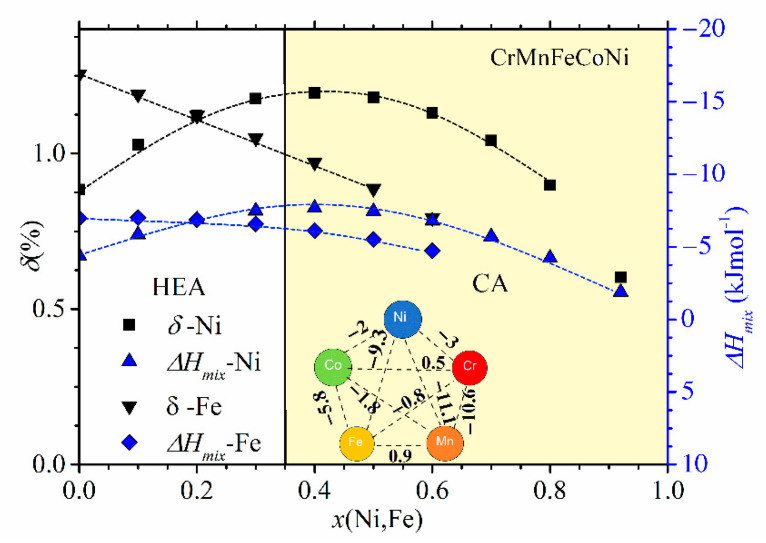
Atomic size mismatch *δ* and mixing enthalpy ∆*H*_mix_ of (CrMnFeCo)_1−x_Ni_x_ and (CrMnCoNi)_1−x_Fe_x_ alloys vs. *x*. The inset: ∆*H*_mix_ between the constituent elements.

**Figure 4:**
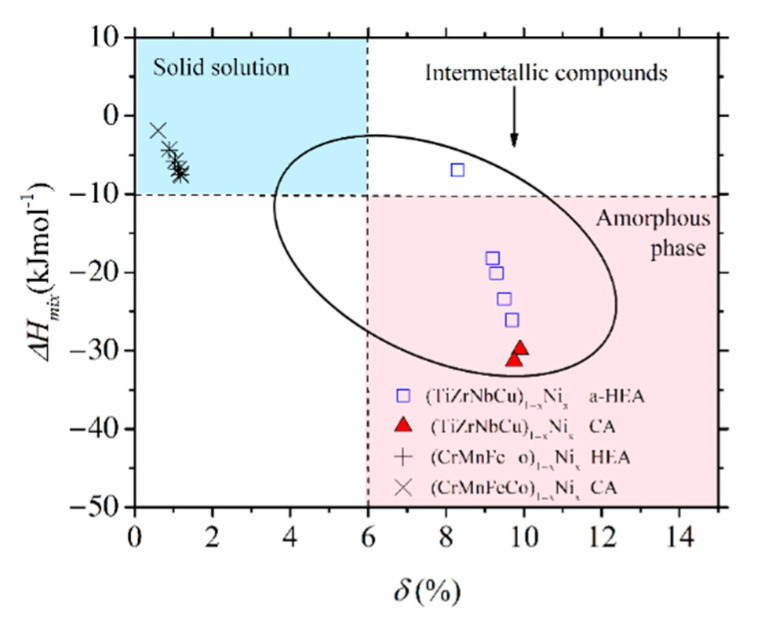
A plot of ∆*H*_mix_ vs. *δ* showing the data for (CrMnFeCo)_1−x_Ni_x_ and (TiZrNbCu)_1−x_Ni_x_ alloys. Data for conventional alloys (CAs) are denoted with different symbols. Black oval encompasses alloys comprising intermetallic compounds.

Despite their limitations and some erroneous predictions (such as the occurrence of an IM in the SS region and an SS in the a-HEA region, e.g., [10,11,12,13,16,23,54,55,56]), as illustrated in Figure 2, Figure 3 and Figure 4, these criteria are useful for a quick comparison of different HEA systems (Figure 4). The variation of thermophysical parameters with the composition within a given alloy system can, on the other hand, provide an insight into the evolution of the properties of this system (Figure 2 and Figure 3, [24,25,52]).

In Figure 2 and Figure 3, we show the compositional variations of selected thermophysical parameters in characteristic quinary TE-TL [24,25] and Cantor-type alloys [45,47], respectively. In the calculation of these parameters, we used standard expressions (see, e.g., [12,13,24,25]), and the input parameters for ∆*H*_mix_ and *δ* in Figure 2 and Figure 3 and their insets were taken from References [71,72], respectively. For simplicity, a rather well-known variation of ∆*S*_conf_ [25], which depends only on the number of alloying components and not on their type [10,11,12,13], is not shown in these figures. In Figure 2, showing the variations of parameters of (TiZrNbCu)_1−x_Ni_x_ and (TiZrNbNi)_1−x_Cu_x_ alloys with *x*, the concentration range of HEAs, *x* ≤ 0.35, is distinguished from that of Ni- or Cu-rich alloys by a different color. The range of values of ∆*H*_mix_ (from −32 to −6.6 kJmol^−1^) and of *δ* (from about 8% to 10%) places our alloys in a standard ∆*H*_mix_−*δ* plot [12] within the region occupied with an IM (*x* = 0 Ni) and a-HEAs (other alloys), which is consistent with our experimental findings. As it can be seen in the inset, small values of ∆*H*_mix_ are the consequence of strong interatomic interactions between TE and TL atoms [71], and a large *δ* is similarly due to the large difference in size between TE and TL atoms. Since a small ∆*H*_mix_ and large *δ* are general features of TE-TL alloys, this facilitates the comparison of the results for our quinary MGs with previous results for properties of similar binary ones [73,74,75].

The comparison of ∆*H*_mix_ and *δ* in Figure 2 shows that both the magnitudes and variations of *δs* are quite similar in the two alloy systems, whereas the corresponding variations of ∆*H*_mix_ are quite different. In (TiZrNbCu)_1−x_Ni_x_ alloys, ∆*H*_mix_ decreases rapidly from −6.6 for *x* = 0 to −32 kJmol^−1^ at *x* = 0.5, whereas in (TiZrNbNi)_1−x_Cu_x_ alloys, ∆*H*_mix_ increases nearly linearly from −28.2 to −15 kJmol^−1^ within the same concentration range. The inset in Figure 2 shows that the values of ∆*H*_mix_ between Cu and Ti, Zr or Nb [71] are about three or more times larger than those between Ni and Ti, Zr or Nb, which probably explains the linear increase in ∆*H*_mix_ with the Cu content. This large reduction in interactions between Cu and TE atoms strongly affects all properties of (TiZrNbNi)_1−x_Cu_x_ alloys. These MGs show an ideal solution behavior [25] which results in linear variations of their properties with *x* such as that depicted in Figure 1. In contrast, stronger interactions of Co and Ni atoms with TE ones (inset in Figure 2) lead to more complex variations of their properties with the concentration [24,26,37,52].

Compositional variations of ∆*H*_mix_ and *δ* in (CrMnFeCo)_1−x_Ni_x_ and (CrMnCoNi)_1−x_Fe_x_ alloys are shown in Figure 3. We selected these two alloy systems because they, as with those shown in Figure 2, exhibit very different variations of their properties with the composition [45,47]. In these alloy systems, an FCC crystalline structure forms over a broad concentration range [45,46,47]. We note that the concentration scale in Figure 3 extends up to *x* = 1 due to the very broad concentration range of a single-phase solid solution with an FCC structure in (CrMnFeCo)_1−x_Ni_x_ [45]. Taking into account that the studied concentration range in Figure 3 is considerably broader than that in Figure 2, the variations of thermophysical parameters in Figure 2 and Figure 3 are qualitatively similar. The variation of ∆*H*_mix_ of (CrMnFeCo)_1−x_Ni_x_ alloys with *x* is qualitatively similar to that in (TiZrNbCu)_1−x_Ni_x_ alloys, whereas that in (CrMnCoNi)_1−x_Fe_x_ alloys is similar to the variation observed in (TiZrNbNi)_1−x_Cu_x_ alloys. As with the quinary TE-TL alloys [24,25,26], different variations of ∆*H*_mix_ with *x* correspond to different compositional variations of their properties [45,46,47]. Some properties of (CrMnCoNi)_1−x_Fe_x_ alloys vary linearly with *x* within the range of FCC SSs [46], which is similar to what is observed in (TiZrNbNi)_1−x_Cu_x_ MGs [25], whereas those in (CrMnFeCo)_1−x_Ni_x_ alloys show a more complex behavior similar to that illustrated in Figure 1. Since the alloying components in a Cantor type of alloy seem to form a common valence band (see PES results in Section 3.4 and [30]), VEC should provide a reasonable approximation for their ES. Accordingly, the VEC criterion for the selection of the crystalline phase in HEAs [60] should describe both the crystalline phases of Cantor alloys and their evolution with the composition. According to this criterion, the alloys with VEC ≥ 8 should have a stable FCC phase. This criterion is consistent with a single FCC phase in (CrMnFeCo)_1−x_Ni_x_ alloys (since VEC ≥ 8 throughout the explored concentration range) but is at variance with the onset of a BCC phase in (CrMnCoNi)_1−x_Fe_x_ alloys for *x* ≥ 0.7 (since in these alloys, VEC = 8 and does not depend on *x*).

Despite the qualitatively similar variations of thermophysical parameters in Figure 2 and Figure 3, the magnitudes of these parameters are quite different. As it could be expected for alloys composed of similar, adjacent elements, the values of *δ* in Figure 3 are small, around 1%, thus about ten times smaller than those in Figure 2. The corresponding ∆*H*_mix_ values are larger than −7.5 kJmol^−1^, thus, on average, several times larger than those in Figure 2. Such relatively large values of ∆*H*_mix_ in Figure 3 result from moderate interatomic interactions between the alloying components [71], as seen in the inset in Figure 3. Indeed, the smallest value of ∆*H*_mix_ among these elements is that between Mn and Ni (−11.1 kJmol^−1^). (The relatively strong interaction between Ni and Mn atoms is reflected in the modest fluctuations in the composition between the dendritic and interdendritic regions in as-cast Cantor-type alloys [45].) As a result of large differences in the magnitudes of thermophysical parameters, Cantor-type alloys and quinary TE-TL alloys occupy very different parts of the standard ∆*H*_mix_ vs. *δ* diagram [9,10,11,12,13]. This is illustrated for (CrMnFeCo)_1−x_Ni_x_ and (TiZrNbCu)_1−x_Ni_x_ alloys in Figure 4: the first alloys are placed in the SS region, characterized by a small *δ* ≤ 6.5% and modest interatomic interactions, −15 kJmol^−1^ ≤ ∆*H*_mix_ ≤ −5 kJmol^−1^ (both consistent with the Hume-Rothery rules [12]), whereas the second alloys are placed in the opposite region of large *δ*s, extending from that occupied by IMs for *x* = 0 to that of a-HEAs for *x* ≥ 0. We note that the ∆*H*_mix_−*δ* diagram, although useful for the classification of HEAs into SS, IM and a-HEA groups, is not associated solely with HEAs. Despite their broad compositional range covering both HEAs and CAs (denoted in Figure 4 with different symbols), the data for all (CrMnFeCo)_1−x_Ni_x_ alloys are neatly grouped within the region of SSs and those of (TiZrNbCu)_1−x_Ni_x_ alloys in the IM/a-HEA region. Moreover, all alloys obeying the Hume-Rothery rules will be placed within the SS region [12], regardless of their composition and the number of alloying components, whereas those in accordance with Inoue´s rules [59] are likely to be placed in the IM or a-HEA region. There are, however, some alloys which can be prepared as MGs, but the final phase of these alloys depends on the preparation and processing conditions [51,53,54,55,56]; thus, the ∆*H*_mix_−*δ* diagram is not sufficient for their classification.

Rather strong interactions of Ni, Co and Cu atoms with TE atoms (inset in Figure 2) and a high melting point of Nb can all affect the distribution of constituents in our TE-TL alloys [53]. Similarly, very different strengths of interatomic interactions between different components of Cantor-type alloys (inset in Figure 3) can produce a somewhat inhomogeneous distribution of elements within these alloys [9]. Since Cantor-type alloys contain large fractions of magnetic elements, their magnetic properties will be strongly affected by their distribution. Accordingly, we performed EDS mapping of the distribution of constituent elements in all as-cast TE-TL MGs and homogenized Cantor-type alloys. As described in Section 2 and previous papers [23,24,25,45,46,47,48,52], elemental mapping was performed on three different areas of each alloy to access the eventual inhomogeneity in the distribution of the constituents caused by the composition variation in different areas of the same sample. Mapping was also used to obtain information about the possible size and shape of such inhomogeneity (e.g., [9,53]). This is illustrated in Supplementary Materials Figures S1 and S2, which show the evolution of the microstructure and elemental distribution (chemical homogeneity) in the as-cast and rolled and annealed (homogenized) (CrMnFeCO)_1−x_Ni_x_ samples with *x* = 0.3 and 0.92, respectively. As previously described in some detail [23,24,25,45,46,47,48,52] and illustrated in Supplementary Materials Figures S1 and S2, the distributions of constituent elements were random down to micrometer scale in all studied alloys. We did not observe any clear correlation or anticorrelation between the distributions of different elements in the elemental mappings. Although these elemental mappings cannot exclude some compositional fluctuations on a nanometric scale, such fluctuations, even if present, are not likely to have any larger effect on the macroscopic (bulk) properties of nonmagnetic TE-TL MGs. However, such fluctuations can affect the magnetism in 3d transition metal alloys, as seen from the very different descriptions of the magnetic state of the Cantor alloy in different papers (e.g., [5,7,13,15]). Further, in all samples, the composition calculated from EDS at different locations was the same within about 1 at. %, which is an indication of their macroscopic homogeneity. Since the average concentrations of all alloys obtained from EDS were within about 1 at. % of the corresponding nominal ones, we will continue to use the nominal compositions in our further analyses.

### 3.2. Thermal Parameters

Thermal parameters are particularly important since they determine the useful temperature range in all alloys (e.g., [8,13,76,77,78]). Further, these parameters are related to the strength of interatomic bonding in an alloy and can also provide an insight into the glass-forming ability (GFA) of alloys that can be vitrified. Unfortunately, thermal analysis of HEAs is frequently ignored, and their thermal parameters are estimated by using the rule of mixtures [8] which often provides erroneous values of these parameters in both c-HEAs (e.g., [45,46,47,78]) and a-HEAs [23,24,25,37]. We note, however, that for some alloy systems, such as those based on refractory elements, the temperature span of commercial DSCs (usually *T* ≤ 1400 °C) may not be sufficient for their complete thermal analysis.

**Figure 5:**
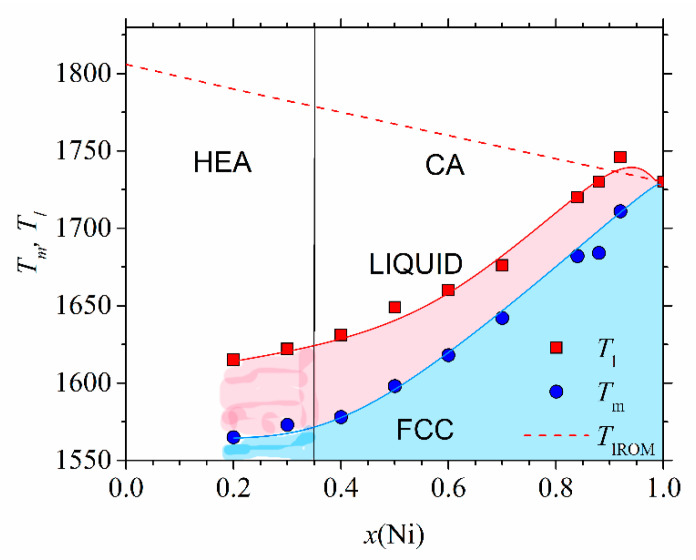
High-temperature phase diagram of (CrMnFeCo)_1−x_Ni_x_ alloys. The region of coexistence of a solid and a liquid is colored magenta. The red dashed line denotes variation of *T*_m_ calculated using the rule of mixtures.

Figure 5 shows the high-temperature part of the experimental phase diagram of (CrMnFeCo)_1−x_Ni_x_ alloys obtained from DSC measurements (similar to those shown in [78]). Only the part of the phase diagram corresponding to alloys with an FCC crystalline structure (*x* ≥ 0.2) is shown. Different colors denote different phases: above *T*_l,_ the alloy is in a liquid state, and in the temperature interval between *T*_l_ and *T*_m_, a coexistence of a solid phase and liquid is established. Below *T*_m,_ all alloys with *x* ≥ 0.02 possess a single-phase FCC structure as verified by using their XRD patterns [45,46]. This part of the phase diagram agrees rather well, both qualitatively and quantitatively, with that calculated by using CALPHAD [46]. Only a small maximum of *T*_l_ around *x* = 0.9 is not reproduced by the calculation. The values of *T*_m_ (dashed line), calculated by using the rule of mixtures, are at variance with the experimental ones. The rule of mixtures predicts a linear decrease in *T*_m_ from 1792 K for *x* = 0.2 towards that of pure Ni, whereas the experimental values increase nonlinearly from 1563 to 1728 K for pure Ni. As it will be seen later, a rapid increase in *T*_m_ for *x* ≥ 0.4 coincides with the onset of ferromagnetism in these alloys. In contrast, the values of *T*_m_ in (CrMnCoNi)_1−x_Fe_x_ alloys increase practically linearly with *x* from 1505 K for *x* = 0 to that of pure Fe (1808 K) [47]. Thus, in both these alloy systems, the thermal stability of CAs (*x* ≥ 0.35) is better than that of HEAs; hence, these HEAs are unlikely to be used at elevated temperatures. We note that the values of *T*_m_ calculated from the rule of mixtures would lead to an opposite, apparently erroneous conclusion on the evolution of thermal stability with the concentration. Indeed, as already noted in Figure 5, the rule of mixtures predicted both incorrect values of *T*_m_ and erroneous concentration dependence. The predictions of the rule of mixtures for *T*_m_ of other Cantor types of alloys [47] were similarly erroneous as those in Figure 5. At lower temperatures, the phase diagram of (CrMnFeCo)_1−x_Ni_x_ alloys [46] and other Cantor types of alloys [47] becomes much more complex, composed of different phases in different temperature and concentration ranges. As a result, prolonged annealing of these initially single-phase alloys, at temperatures below about 900 K, results in precipitation of other phases within the matrix [9,13], which further limits their applicability at higher temperatures.

**Figure 6:**
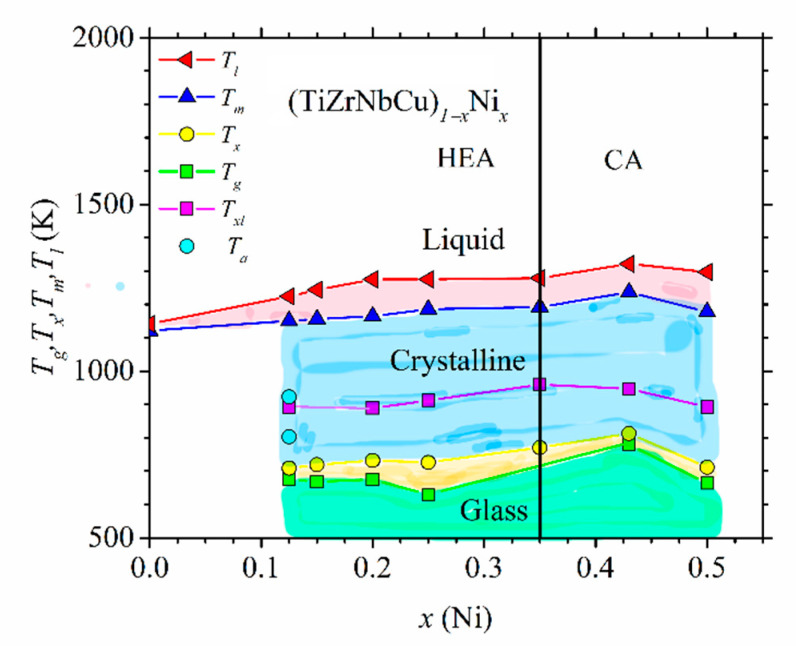
Thermal parameters of (TiZrNbCu)_1−x_Ni_x_ alloys vs. *x*. *T_x_* and *T_xl_* denote temperatures of the first and last crystallization events, respectively. *T_a_*s denotes annealing temperatures of the alloy with *x* = 0.125. Different phases are differently colored.

Similarly, DSC/DTA analysis [23,24,25,37,52] was used to determine thermal parameters of (TiZrNbCu)_1−x_Co_x_, (TiZrNbCu)_1−x_Ni_x_ and (TiZrNbCu)_1−x_Cu_x_ alloys exhibiting an amorphous XRD pattern after melt spinning. Thermal analysis of amorphous alloys enables, in addition to the determination of *T*_m_ and *T*_l_, the extraction of the glass transition (*T*_g_) and crystallization (*T*_x_) temperatures. These thermal parameters form the non-equilibrium phase diagram of glass-forming systems [25,52]. In Figure 6, we show such a diagram for (TiZrNbCu)_1−x_Ni_x_ alloys.

As in Figure 5, different phases are colored with different colors. Here, in addition to three phases appearing in Figure 5, the diagram contains the amorphous state below *T*_g_ and a supercooled liquid state situated between *T_g_* and *T_x_*. All quinary TE-TL MGs studied by us [23,24,25,37,52] showed complex crystallization patterns reflected in three or more exothermic maxima spread over a broad temperature range. These consecutive crystallizations are consistent with a strong but quantitatively different bonding tendency between different TE and TL atoms inferred from their thermophysical parameters shown in the inset in Figure 2. Due to this, in Figure 6, *T_x_* denotes the onset of the first crystallization event, and *T_xl_* denotes the temperature of the exothermic maximum corresponding to the last crystallization event appearing in the corresponding DSC trace. Rather complex crystallization processes in quinary TE-TL MGs will make future studies of the evolution of crystallization products with the composition and temperature much more complicated than those for the corresponding binary MGs [73,76,77]. Thus far, we have crystallized only an MG with *x* = 0.125, and the corresponding annealing temperatures are denoted by *T_a_* in Figure 6. The selected values of *T_a_* are above *T_x_* and *T_xl_*.

Thermal parameters associated with the thermal stability of different phases and the interatomic bonding, such as *T_x_* and *T*_m_, increase somewhat with the Ni content in Figure 6. Thus, as in the Cantor-type alloys shown in Figure 5, the thermal stability of these alloys in the HEA concentration range is inferior to that of the corresponding CAs. Further, as in crystalline (CrMnFeCo)_1−x_Ni_x_ alloys (Figure 5) and other quinary TE-TL alloys [25,52], the values of *T*_m_ calculated by using the rule of mixtures are at variance with those in Figure 6. The rule of mixtures predicts a linear decrease in *T*_m_ from 2041 for *x* = 0 to 1883 K for *x* = 0.5, whereas the experimental values increase from 1121 to 1179 K over the same concentration range. The observed strong deviation of experimental values of *T*_m_ from those calculated by using the rule of mixtures is probably associated with a strong bonding tendency between alloying elements (inset in Figure 2) and with the local atomic arrangements around Ti and Zr atoms which are different from those in the stable phases of the corresponding pure metals [23,24,25,52,66]. By using the experimental values of *T*_m_, we can compare the contributions to the free energy from ∆*H*_mix_ (Figure 2) and ∆*S*_conf_ *T*_m_. As is common in TE-TL alloys [23,25,52,53,54,66], ∆*H*_mix_ outweighs ∆*S*_conf_ *T*_m_ due to the strong interatomic bonding in all our alloys containing Ni (*x* ≥ 0.125). Since our as-cast alloy TiZrNbCu, in which ∆*S*_conf_ *T*_m_ (12.84 kJ/mole) considerably outweighs ∆*H*_mix_ (6.6 kJ/mole, Figure 2), ∆*S*_conf_ *T*_m_/∆*H*_mix_ = 1.95, was multiphase (IM) [23], it seems that in our alloys, configurational entropy has limited influence on the formation of either an SS or an amorphous phase.

The ratio *Ω* = ∆*S*_conf_ *T*_m_/∆*H*_mix_, where *T*_m_ is calculated by using the rule of mixtures, is a commonly accepted criterion for the phase formation of HEAs [12,79]. In particular, single-phase SSs are expected for *Ω* ≥ 1.11, whereas a-HEAs are situated below that value in the *Ω* vs. *δ* plot. Since the values of *T*_m_ calculated by using the rule of mixtures are much larger than the experimental ones, the corresponding values of Ω place all our alloys above the region occupied by amorphous alloys in the *Ω* vs. *δ* plot [79]. (As already noted in Section 3.1 and shown in Figure 4, our alloys are correctly placed in the IM and a-HEA regions of the ∆*H*_mix_ vs. *δ* plot [12].) This indicates that some erroneous predictions of the phase of HEAs obtained by using the Ω criterion in both a- and c-HEAs (e.g., [80]) may arise from the use of the calculated instead of the observed value of *T*_m_ in the definition of this criterion [79]. This again emphasizes the importance of measurements of the thermal parameters of HEAs and other CCAs.

The increase in thermal parameters with *x* in Figure 6 is the usual behavior of binary and ternary TE-TL MGs [73,81,82,83,84,85], which was recently also observed in quinary TE-TL MGs [24,25,52]. Such behaviors are usually accompanied by a simultaneous enhancement of the mechanical properties and the Debye temperatures with increasing *T*_L_ content, all of which support an increase in the strength of interatomic bonding [24,52,66,81,82,83,84]. However, the variations of thermal parameters with *x* in our alloys (Figure 6) are somewhat different from those in binary [66] and quinary [25] TE-Cu alloys. In TE-Cu MGs, quasi-linear variations of all thermodynamic properties with the Cu content indicate an ideal solution behavior, and thus a smooth transition from HEAs to Cu-rich conventional alloys in (TiZrNbNi)_1−x_Cu_x_ MGs [25]. In our alloys, *T*_x_, *T*_m_ and *T*_g_ (except for the value at *x* = 0.25) increase linearly with *x* for *x* ≤ 0.43 and then decrease a little at *x* = 0.5 (Figure 6). This change in the variations of thermal parameters at *x* = 0.43 coincides with that observed in all studied properties of (TiZrNbCu)_1−x_Ni_x_ MGs, including the atomic short-range order (SRO), magnetic and mechanical properties [24], electronic transport properties and ES [26]. Thus, in these alloys, the transition from HEAs to Ni-rich conventional alloys is accompanied by a change in all intrinsic properties. However, since in (TiZrNbCu)_1−x_Co_x_ MGs [52], a change in properties seems to occur around *x* = 0.25, thus deep within the HEA concentration range, the change in properties of these alloy systems with the composition is not caused by the transition from HEAs to CAs; instead, it merely reflects the evolution of their ES with *x*.

Next, we use the results for the thermal parameters from Figure 6 in order to discuss a well-known discrepancy between the relatively good thermal stability of a-HEAs and their modest GFA (e.g., [23,63]). It has been proposed [63] that a higher *T*_x_ of the equiatomic TiZrCuNiBe a-HEA compared to that of a benchmark glass Vitreloy 1 (Zr_41.2_Ti_13_._8_Cu_12.5_Ni_19_Be_22.5_) results from sluggish crystallization kinetics in the a-HEA. However, as seen in Figure 6, the higher *T*_L_ (Cu + Ni) content in the a-HEA probably also contributes to its better thermal stability. Further, the vicinity of the composition of Vitreloy 1 to that of a stable intermetallic compound composed of these elements (such as Zr_2_(Cu,Ni) which precipitates first on annealing of Vitreloy 1) probably also compromises its thermal stability in respect to the HEA with a composition far from that of any stable IM composed of the same elements [23]. As noted recently [52], the discrepancy between good thermal stability and a modest GFA of a-HEAs, and more generally quinary TE-TL MGs, can be influenced not only by the proposed different diffusion mechanisms in the solid and liquid a-HEAs [63] but also by complex crystallization patterns in these MGs. As seen in Figure 6, the composition dependence of the temperatures of the first (*T*_x_) and the last (*T*_xl_) crystallization event (which affect the stability of the glass and melt, respectively) can be very different. In particular, if in these alloys, rather stable crystalline compounds form during the last crystallization event around *T*_xl_, their GFA may be compromised since it could be difficult to avoid crystallization during insufficiently rapid cooling of the melt. (Alternatively, a small amount of some very stable phase may considerably increase the liquidus temperature, thus affecting the magnitudes of GFA criteria containing *T*_l_, but without compromising the actual GFA.) Since, the thermal stability of the same MG is associated with the separate first crystallization event (occurring around *T*_x_), it may remain high, regardless of its low GFA. This complex crystallization behavior probably reduces the reliability of criteria for predicting GFA based on thermal parameters in multicomponent TE-TL MGs [25,52,63,86,87]. Such problems almost do not exist in binary and some ternary TE-TL MGs, since their DSC traces usually show a single exothermic maximum; thus, *T*_x_ controls both their thermal stability and GFA [66,73,81,82,83,84,85]. Accordingly, their GFA is usually well described with criteria based on thermal parameters [32,33,66,76,77,81,82,83,84,85].

**Figure 7:**
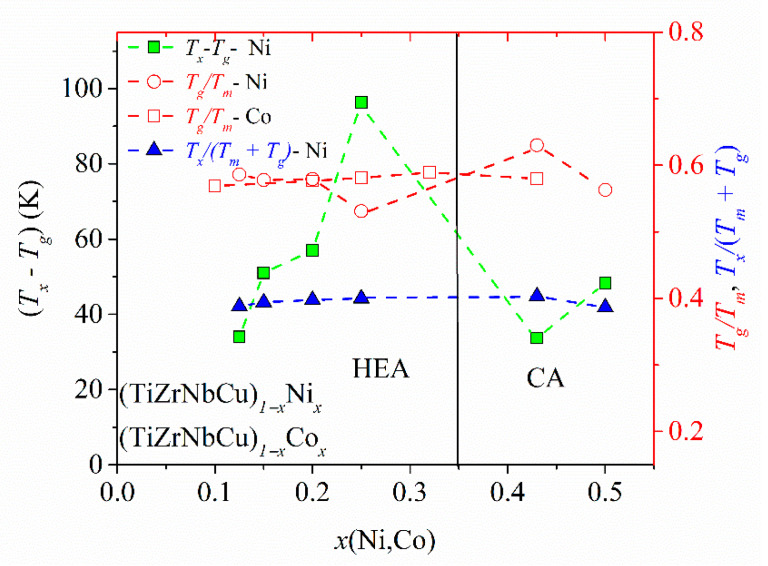
Glass-forming ability parameters of (TiZrNbCu)_1−x_Ni_x_ and (TiZrNbNi)_1−x_Co_x_ alloys vs. *x*.

The variation of the three most common criteria for GFA: the reduced glass transition temperature, *T*_rg_ = *T*_g_/*T*_l_, [88], the *γ* criterion for GFA, *γ* = *T*_x_/(*T*_g_ + *T*_l_) [87], and the width of the supercooled liquid range, ∆ *T*_x_ = *T*_x_ − *T*_g_ [59], with the composition in (TiZrNbNi)_1−x_Cu_x_ and (TiZrNbCu)_1−x_Co_x_ alloys was previously reported [25,52]. In both systems, an enhancement of *T*_l_ at the equiatomic composition, *x* = 0.2, led to a local minimum in the values of *T*_rg_ and *γ*, which would imply a low GFA since, in all three criteria, large values correspond to a high GFA. However, an experimental study of the GFA in (TiZrNbNi)_1−x_Cu_x_ alloys with *x* ≤ 0.35 [25] showed the largest GFA around *x* = 0.2, in contrast to the predictions of *T*_rg_ and *γ*. As seen in Figure 6, *T*_l_ of the TiZrNbCuNi alloy is also somewhat larger than that at neighboring compositions, which would lead to low values of *T*_rg_ and *γ*, and thus the erroneous prediction of a low GFA of the same alloy which showed the best GFA in the (TiZrNbNi)_1−x_Cu_x_ system [25]. Since the values of *T*_m_ did not show such anomalies at *x* = 0.2 [25,52], in Figure 7, we inserted *T*_m_ instead of *T*_l_ into the denominators of the expressions for *T*_rg_ and *γ*. This change removed the minima of *T*_rg_ in both (TiZrNbCu)_1−x_Ni_x_ and (TiZrNbCu)_1−x_Co_x_ alloys (Figure 7). Figure 7 also shows the variation of ∆*T*_x_ with the composition in (TiZrNbCu)_1−x_Ni_x_ alloys, exhibiting a large maximum around *x* = 0.25. A large ∆*T*_x_ is important for the application of MGs [89] and has been found to correlate quite well with the GFA in several a-HEAs (e.g., [25,63]) including that in Figure 7. As already noted [25,52], the rather low values of *T*_rg_ and *γ* in the studied alloys are consistent with their modest GFA. Indeed, in contrast to the similar TiZrHfCuNi alloy [1], none of these alloys formed a bulk metallic glass [25]. This could be associated with somewhat weaker interatomic interactions and a smaller mismatch of the atomic size in alloys containing Nb than these in alloys containing Hf [59].

**Figure 8:**
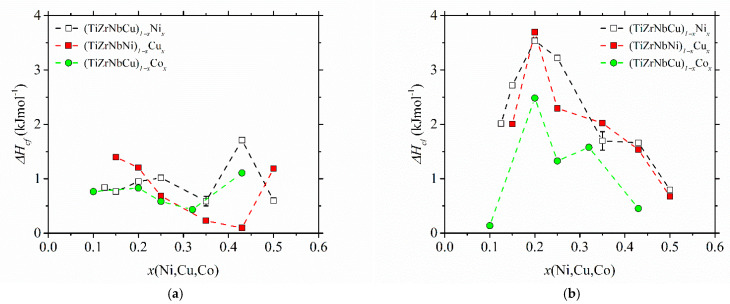
Crystallization enthalpies of (TiZrNbNi)_1−x_Cu_x_, (TiZrNbCu)_1−x_Ni_x_ and (TiZrNbNi)_1−x_Co_x_ alloys vs. *x*: (**a**) corresponding to the first crystallization event, ∆*H*_cf_, and (**b**) corresponding to the last crystallization event, ∆*H*_cl_.

The variations of the enthalpy change in crystallization, ∆*H*_c_, with the composition in the studied alloys are shown in Figure 8. ∆*H*_c_ values were determined from the crystallization DSC peak areas. Due to some partially overlapping peaks [25,37,52], their values are not that accurate. In cases of overlapping peaks (e.g., the first peak in (TiZrNbCu)_0.57_Co_0.43_ in [52]), we arbitrarily assigned half of the total ∆*H*_c_ to each of the two crystallization events. However, the reproducibility of measurements was quite good, as seen from the error bars based on measurements of two different samples for alloy (TiZrNbCu)_0.65_Ni_0.35_. The measurements of ∆*H*_c_, which shows the change in free energy between the competing phases [73], are apparently important both for understanding thermal stability and the GFA [32,33] in glass-forming alloys. It is therefore surprising that, to our knowledge, no previous results for ∆*H*_c_ of a-HEAs exist. Comparing the results for the change in enthalpy associated with the first crystallization event (∆*H*_cf_ in Figure 8a) with those for the last (final) crystallization event (∆*H*_cl_ in Figure 8b), we note that both the compositional variations and magnitudes of the two quantities are quite different. The magnitudes of ∆*H*_cf_ are fairly small, and their compositional variations do not show any obvious tendency. Their increase at *x* = 0.43 in alloys with variable Ni and Co contents may be affected by the overlap of the two peaks. The values of ∆*H*_cl_ are considerably larger, and all show a pronounced maximum centered around an equiatomic composition, both indicating the formation of a relatively stable phase during the last crystallization around this composition in all studied alloys. Thus, these results provide some support to the former discussion of the evolution of thermal stability, compositional variation of thermal parameters and GFA of the studied alloys. However, for a more detailed insight into these issues, XRD analysis of the phases associated with each crystallization event is required.

### 3.3. Atomic Structure

Comprehensive studies of the atomic arrangements and their evolution with the composition in Cantor-type alloys possessing a single FCC phase [45,47] and quinary MGs composed of Ti, Zr, Nb, Cu and Ni or Co have already been reported [24,25,37,52], or are in preparation for submission [62]. For completeness, we will briefly describe the main features of these results and then compare the results for two types of alloys. Some new results will also be shown.

The lattice parameters, *a*_FCC_, and corresponding average atomic volumes, *V*_FCC_ = *a*_FCC_^3^/4, of Cantor-type alloys with a variable Mn, Fe or Co content vary linearly with the composition within the range of stability of a single FCC phase in homogenized samples [45,47]. Hence, the same variation extends from the HEA to the Mn-, Fe- or Co-rich concentration range. (Although this concentration range extends to *x* = 0.5 for Mn, Fe and Co, the results for two-phase alloys indicate that a linear variation of *V*_FCC_ may extend up to *x* = 0.7 and 0.9 for Fe and Co, respectively [47].) The slopes of the linear variations of *V*_FCC_s and ∆*V*_FCC_/∆*x* are negative for principal alloying elements Fe and Co, zero for Cr (*x* ≤ 0.3) and large and positive for Mn [47]. These slopes probably reflect both the atomic size mismatch and the strength of the interatomic bonding (inset in Figure 3) in each alloy system. Accordingly, these variations of *V*_FCC_s strongly affect their mechanical properties [47,48]. (CrMnFeCo)_1−x_Ni_x_ alloys are exceptional among these alloys in that their *a*_FCC_ hardly changes until *x* ≤ 0.5 and then decreases linearly with x according to Vegard´s law to that of pure Ni [45]. This change in the variation of *a*_FCC_ in the Ni-rich concentration region seems to be associated with both the compositional variation of the alloys’ thermal stability (Figure 5) and that of their mechanical properties [45,47,48].

Analysis of the atomic arrangements in amorphous solids is more involved, and the results of this type of analysis are less detailed than those for crystalline solids [90,91]. In principle, analysis of the XRD pattern of amorphous alloys can provide the average distances between the nearest neighbor atoms [92], *d*, but these are not that accurate and may also not be reliable [93]. High-energy XRD (HEXRD) can provide the radial distribution function, *R*(*r*), and pair distribution functions, PDF, from which more accurate and reliable interatomic distances and numbers of atoms in neighboring shells around an atom (coordination numbers, *N*) can be calculated. However, neither simple XRD nor HEXRD can provide direct insight into the local atomic arrangements in an amorphous alloy.

**Figure 9:**
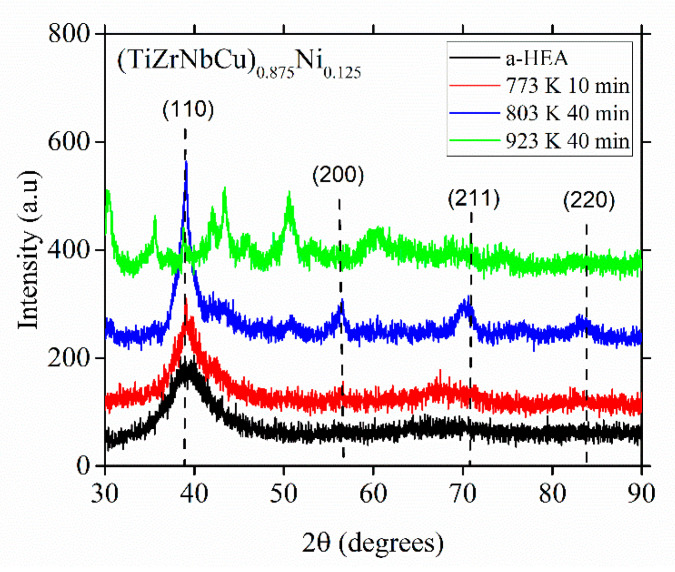
XRD patterns of as-cast (a-HEA) TiZrNbCu)_0.875_Ni_0.125_ sample and TiZrNbCu)_0.875_Ni_0.125_ samples annealed at specified temperatures. Vertical dashed lines denote positions of maxima in β-Ti.

As seen in Figure 9, an insight into a probable local atomic arrangement in the glassy alloy can be obtained from the product(s) of the primary crystallization in that alloy. The XRD patterns in this figure show how the atomic structure of (TiZrNbCu)_0.875_Ni_0.125_ evolves upon annealing at different temperatures above *T*_x_ marked in Figure 6. We note that the primary crystallization produces a dominant, fine-grained BCC crystal structure with a lattice parameter close to that of β-titanium (vertical dashed lines), around 0.326 nm [23]. This BCC phase is, however, metastable since the XRD pattern obtained after crystallization at a temperature surpassing the last crystallization event (Figure 6) bears no resemblance to that for the primary crystallization, Figure 9.

In these alloys, the good agreement between the calculated mass density obtained by assuming a BCC atomic structure and the measured one [23] also provides strong support for a BCC-like local atomic structure. As discussed in some detail elsewhere [52], there are several reasons which support BCC-like local atomic arrangements in the studied glassy alloys composed of Ti, Zr, Nb, Cu and Ni or Co and probably all other a-HEAs composed of TE and TL atoms (e.g., [51,54,56]). We note that the large difference between the sizes of TE and TL atoms [72] and the corresponding atomic size mismatch, *δ*, (Figure 2) also make the formation of a BCC-like local atomic arrangement in TE-TL alloys more likely than the FCC one [12,53,54,56]. Indeed, in all ∆*H*_mix_ vs. *δ* diagrams, the single-phase BCC alloys are situated at larger values of *δ* than those with an FCC crystalline structure [10,11,12,13]. The strong bonding tendency between the TE and TL atoms may also favor the formation of a BCC-like atomic arrangement since the ordering of different atoms seems easier to achieve on a BCC than on an FCC atomic structure [51,54].

**Figure 10:**
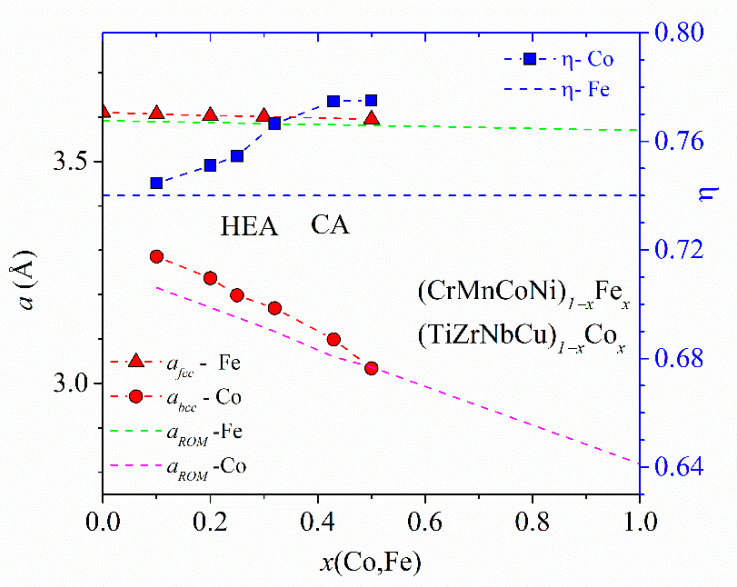
Lattice parameters *a* and the corresponding atomic packing fractions *η*_a_ of (CrMnCoNi)_1−x_Fe_x_ and (TiZrNbNi)_1−x_Co_x_ alloys vs. *x*. Green and magenta dashed lines denote variations of *a* calculated by using the rule of mixtures.

Accordingly, we used the values of *d*, obtained both from the first maximum of the XRD pattern [91] and from *R*(*r*) [89,90], to calculate the average BCC-like lattice parameters, *a*_BCC_ = 2 *d*/3^0.5^, and the corresponding average atomic volume, *V*_BCC_ = *a*_BCC_^3^/2, of all studied alloys [24,25,52,61]. From these average atomic volumes, we calculated the mass densities and the average local atomic packing fractions (APFs, [24,25,52]), *η*_a_, which depend on the local atomic arrangements, whereas from *R*(*r*), we calculated the corresponding coordination number, *N*. Two types of compositional variations of these parameters were observed. In (TiZrNbNi)_1−x_Cu_x_ MGs, showing an ideal solution behavior [25], both *a*_BCC_ and *V*_BCC_ decrease linearly with *x*, whereas the average APF (around 0.75) and *N* (around 13) practically do not change with *x* (implying no changes in the local atomic arrangements) [25]. As illustrated in Figure 10, qualitatively, the same behavior of these parameters is observed in FCC (CrMnCoNi)_1−x_Fe_x_ alloys [47]: *a*_FCC_ is very close to that calculated by using Vegard´s law and decreases linearly with *x*, whereas the APF is constant at around 0.74 (which is consistent with the close-packed FCC structure composed of atoms of a similar size).

The other type of behavior of parameters associated with the atomic structures observed in (TiZrNbCu)_1−x_Ni_x_ and (TiZrNbCu)_1−x_Co_x_ MGs [24,52] can be broadly compared to that in (CrMnFeCo)_1−x_Ni_x_ FCC alloys [45]. In these alloy systems, *d* and *V*_BCC_ slightly changed their variations with *x* around some concentrations specific to a given alloy system. These concentrations were around 0.43 and 0.25 in alloys with a variable Ni or Co content, respectively. Around these concentrations, APFs and *N* exhibited a rapid change [24,52,62]. This is illustrated in Figure 10, which shows the variations of *a*_BCC_ and the APF with the concentration in (TiZrNbCu)_1−x_Co_x_ MGs (including the results for the new alloy with *x* = 0.5). A very large decrease in *a*_BCC_ with *x* compared to that of an FCC structure is due to a large atomic size mismatch in TE-TL MGs. A sudden increase in the APF around *x* = 0.25 is accompanied with a similar increase in *N* [61] at the same concentration. A similar change in the APF and *N* was observed in (TiZrNbCu)_1−x_Ni_x_ MGs, but around *x* = 0.43 [24,62]. Thus, in the studied TE-TL MGs, the crossover concentrations increase going from Co to Ni. This is different from the behavior observed in Cantor-type alloys [47], where alloys with a variable Co content did not seem to show any change in parameters associated with the atomic structure throughout the explored concentration range, *x* ≤ 0.7. As already noted, a change in variations of parameters associated with the atomic structure is accompanied by a change in the intrinsic properties of the same alloys, both in Cantor-type FCC alloys [45,47,48] and quinary TE-TL MGs [24,26,52]. In the next section, a brief discussion of differences in the ES of two types of alloys is presented.

### 3.4. Electronic Structure and Physical Properties

As already pointed out in the Introduction, the ES determines all intrinsic properties of materials [44]. Thus, detailed knowledge of the ES is necessary both for understanding the properties of materials and for the design of new materials with desired characteristics. It has been known for some time that the ES controls the atomic structure and properties in dilute Al-based alloys with 3d transition metals [94]. However, a study of simple binary TE-TL MGs [66] showed that the relationship between the ES and some properties such as the electronic transport properties is not necessarily simple. It is a notorious fact that electronic transport properties are quite simple to measure but fairly difficult to interpret.

The relationship between the ES and intrinsic properties that are barely affected by the exact preparation and/or post-processing conditions is particularly simple in TE-TL MGs [66,67,74,75,81,82,83,84,95]. Early ultraviolet photoemission spectroscopy (UPS) and X-ray photoemission spectroscopy (XPS) studies of these alloys [96,97,98,99] revealed a split valence band (VB) structure with the full or nearly full d-sub-bands of TL elements positioned well below the Fermi level (*E*_F_). With the sub-band of TL remaining well below *E*_F_, there is an approximately linear variation of most intrinsic properties of these MGs with the TL content over a broad concentration range [66,67,74,75,81,82,83,84,95,100]. Further, it was shown that the split band shape of the VB of TE-TL alloys also applies to crystalline alloys [99] and is rather insensitive to the number of alloying components [97]. In these alloys, the d-band shift from the Fermi level of 3d transition metals increases with the increasing atomic number of 3d elements, i.e., the binding energy *E*_B_ increases as we go from Mn to Cu [97]. Additionally, a decrease in *E*_B_ is observed for a given TL when its relative content in the alloy is increased [96]. The magnitude of the decrease in *E*_B_ depends on TL and increases as we go from Cu to Mn [96].

**Figure 11:**
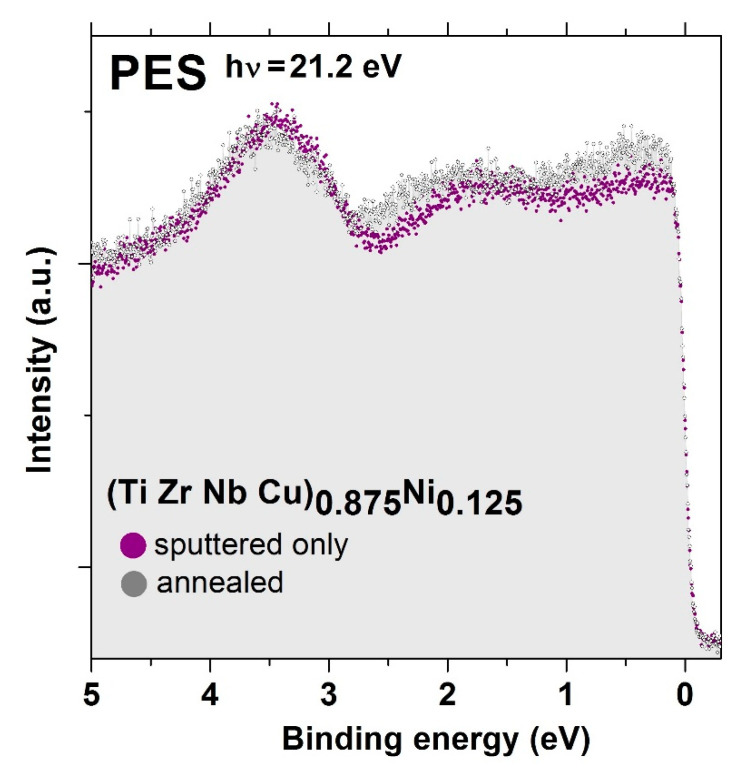
Ultraviolet photoemission spectra of as-cast (denoted sputtered only) and annealed (TiZrNbCu)_0.875_Ni_0.125_ samples. The annealing conditions correspond to those of the uppermost XRD pattern in Figure 9.

**Figure 12:**
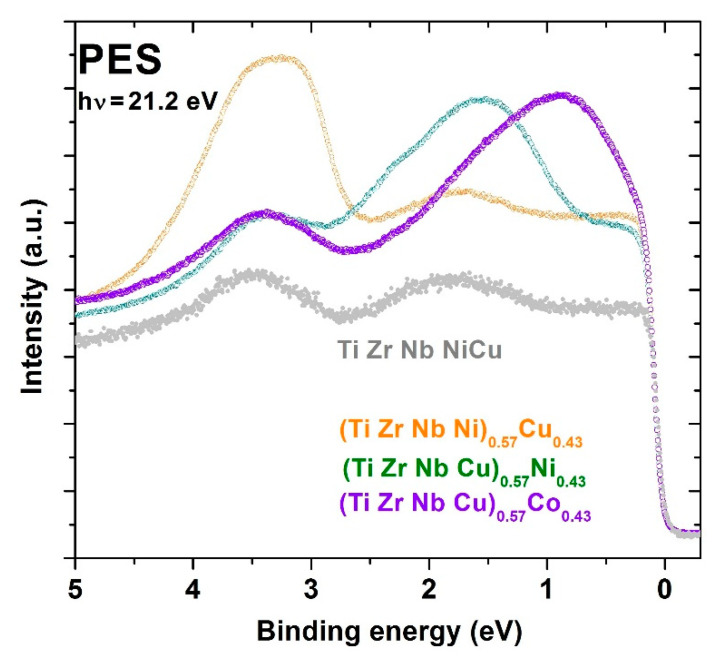
Photoemission spectra of TiZrNbCuNi MG and (TiZrNbNi)_1−x_Cu_x_, (TiZrNbCu)_1−x_Ni_x_ and (TiZrNbNi)_1−x_Co_x_ MGs with *x* = 0.43. Large peaks in alloys with *x* = 0.43 correspond to the 3d states of Cu, Ni and Co.

As seen in Figure 11 and Figure 12, qualitatively, the same behavior of UPS spectra and *E*_B_ was also recently observed in quinary TE-TL MGs studied by us [22,24,25,26].

Figure 11 shows the UPS spectra of the (TiZrNbCu)_0.875_Ni_0.125_ alloy in its (i) as-cast state, amorphous state (denoted by “sputtered only” as a reference to the surface cleaning performed by accelerated Ar ions) and (ii) after crystallization (denoted “annealed”), corresponding to conditions associated with the uppermost XRD pattern of Figure 9. Having in mind the effect of the photon energy-dependent photoemission cross-section [98], these spectra reflect the variation of the electronic density of states (DOS) within the VB. Further, due to the generally low contribution of sp-bands to the photoemission intensity, these spectra largely reflect the DOS of d electrons [24]. The spectra in Figure 11 confirm that crystallization has little influence on the ES of TE-TL MGs, as the variation of the DOS within the VB remains virtually unchanged upon crystallization, and a split band shape of the DOS is retained despite the chemical complexity of the studied alloy. We note three distinct features in both spectra in Figure 11: the maximum around 3.5 eV below the *E*_F_, which we assigned to the 3d states of Cu [25,96,97], and two shallow humps centered around 1.8 eV and close to the *E*_F_, probably related to the d states of Ni and TEs (Ti, Zr and, to a somewhat lesser extent, Nb), respectively [24]. The poorly resolved 3d band maximum of Ni in our alloy is probably due to the nearby maximum of the Nb 4d band [24] as well as the generally very large photoemission cross-sections of TEs at low photon energies [98]. To obtain more accurate information on the position and contribution of the 3d bands of Ni and other TL elements to the DOS of this and other studied alloy systems, XPS measurements using a higher photon energy are required. It is remarkable and probably specific to TE-TL alloys and their GFA [32,33] that a change from an amorphous to a complex crystalline structure (Figure 9) has so little influence on the shape of the UPS spectrum.

Figure 12 shows UPS spectra for three quinary MGs with the same fractions of Co, Ni or Cu, *x* = 0.43, and thus with a concentration within the CA concentration range. For comparison, the UPS spectrum for the equiatomic a-HEA TiZrNbCuNi (*x* = 0.2 [24,25]) is also shown in this figure. Hence, spectra in Figure 12 show the evolution of the DOS of d electrons from the TL components in these alloys when crossing from the HEA to the CA concentration range. Notice that the peak corresponding to the Cu d-band shifts only slightly towards *E*_F_ on increasing *x*, whereas that corresponding to the Ni d-band experiences a considerable shift from about 1.85 to 1.6 eV. However, with an increasing fraction of Co from *x* = 0.2 [52] to 0.43, the peak corresponding to the Co 3d band strongly shifts from 1.3 to 0.8 eV. Thus, despite some uncertainty in the actual position and shape of the peaks associated with the d states of Co and Ni caused by the low energy of the employed photons [97], these spectra clearly show that in MGs with a sufficiently high Co or Ni content, the band crossing, as observed in Zr-Co and Zr-Ni MGs [74,75], takes place at an elevated *x* > 0.43. This band crossing, involving a change in the DOS at *E*_F_, *N*(*E*_F_), dominated, in one case, by d electrons of TE elements and, in another case, by those of TL, results in a change in all intrinsic properties of the given alloys. Figure 12 shows that this crossover concentration [74], *x*_c_, is probably lower for TL = Co [52] than for Ni [24,26], and that in alloys with a variable Cu content [25], showing an ideal solution behavior, this crossover may occur at pure Cu, *x*_c_ = 1, only. More quantitative insight into the approach to the band crossing can be obtained from the variation of *N*(*E*_F_) with the composition, calculated from the LTSH (e.g., [24,26]).

**Figure 13:**
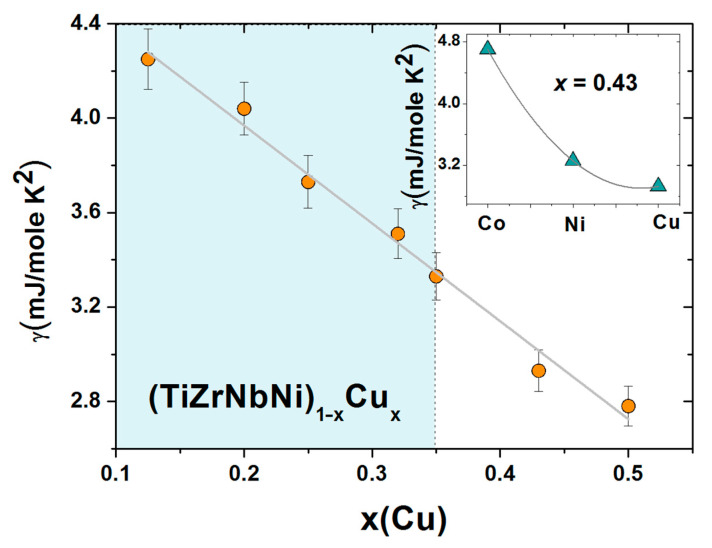
Sommerfeld coefficient *γ* of (TiZrNbNi)_1−x_Cu_x_ MGs vs. *x*. The inset: *γ* of alloys with *x* = 0.43 Co, Ni and Cu.

As previously explained [22,23,24], a combination of the results from PES and LTSH with those from ab initio (*ai*) calculations (which, in addition to the total DOS, also provide information on the contribution of each alloying element to the DOS, pDOS) is required to fully comprehend the ES of an alloy. However, in alloys exhibiting a split band DOS, the experimental results from PES and LTSH may be sufficient to reach that goal, provided that the samples are probed by PES with different photon energies [98]. The use of different photon energies is essential for reliable separation of the contributions of the states of the TL and TE components to the total DOS. This is particularly important when the separation in the energy between these contributions becomes relatively small (see Figure 12). In the simplest case, such as that of (TiZrNbNi)_1−x_Cu_x_ MGs, in which the d states of Cu are well below *E*_F_ and their position hardly changes with *x* (Figure 10 and Figure 12 in Reference [25]), the LTSH measurements are sufficient to determine the evolution of the ES and the intrinsic properties with the composition. New results [101] for the variation of the Sommerfeld coefficient of the linear term of LTSH [24,26], *γ* = π^2^ *k*_B_^2^ *N*(*E*_F_)/3, where *k*_B_ is the Boltzmann constant, with the composition of these alloys are shown in Figure 13. This figure shows that *γ* decreases linearly with increasing *x* over a broad composition range, covering both the HEA (light blue color) and CA concentrations, and extrapolates close to that of pure Cu, *γ* = 0.69 mJ/mole K^2^ [102], for *x* = 1. This variation is probably the best evidence for the ideal solution behavior which marks all properties of this alloy system from the parameters associated with the atomic structure [25] to the magnetic and mechanical parameters, which will be addressed later in the next section. The ideal solution behavior in these chemically complex MGs is likely, as in binary TE-Cu MGs [66,95], caused by the moderate bonding tendency between the TE and Cu atoms (Figure 2) and the nonmagnetic nature of Cu.

As previously discussed in some detail [24,26,52], the variations of *γ* in (TiZrNbCu)_1−x_Co_x_ and (TiZrNbCu)_1−x_Ni_x_ MGs, which are chemically very similar, deviate from the ideal solution behavior at elevated values of *x*. This is associated with the smaller and concentration-dependent values of *E*_B_ in these alloys, which results in the presence of the d states of Co or Ni at *E*_F_ at an elevated *x*. In the inset of Figure 13, we illustrate this effect by comparing the values of *γ* for the alloys with *x* = 0.43 of Co, Ni or Cu. The very strong increase in *γ* for Co, in respect to that for Ni, may, in addition to the enhanced presence of d states of Co at *E*_F_, compared to Ni (Figure 12), also be affected by the onset of electronic correlations, signaling the vicinity of a ferromagnetic percolation threshold around *x* = 0.5 of Co [52]. Since the ES, and often *N*(*E*_F_) alone [44], determines the intrinsic properties of alloys, the variations of *γ*, going from Co to Cu, will be reflected in the variations of other properties of these alloys.

The LTSH measurements were also used to deduce the individual contributions of each TE to *N*(*E*_F_). To this end, we analyzed the values of *γ* for three MGs with an enlarged content of the selected TE at the expense of the other two TEs while keeping the content of TLs the same, e.g., Ti_0.3_Zr_0.15_Nb_0.15_Ni_0.2_Cu_0.2_. The sample with an enhanced Ti content showed the largest *γ* = 4.14 mJ/mole K^2^, which is about 2.5% larger than that of the equiatomic alloy (*x* = 0.2) in Figure 13. This increase in *γ* may arise from the somewhat narrower VB of Ti as compared to that of Zr and Nb, but since *γ* reflects the dressed DOS at *E*_F_, knowledge of the electron–phonon coupling strength [26] is required to gain a more detailed insight into the nature of the observed enhancement of *γ*. The values of *γ* for alloys with an enhanced (0.3) Zr or Nb content were nearly the same and around 2.8% lower than that of the equiatomic alloy, possibly due to the somewhat lower Ti content and wider VBs of Zr and Nb than that of Ti. Accordingly, as in binary and ternary TE-TL MGs [96,97], the d states of Ti contribute slightly more to *N*(*E*_F_) than those of Zr or Nb. The planned UPS and XPS studies of these alloys will probably provide a more detailed insight into the individual contributions of the d states of TEs to the DOS of these MGs.

As seen from the former discussion, the split band structure of the VB in the TE-TL alloys (both crystalline and amorphous, Figure 11) has important consequences on the compositional variation of their properties. However, this also affects the validity of approximate descriptions of the ES, such as those in terms of the average VEC [44], in these alloys [67]. The average VEC quite often provides a reasonable description of the variations of some properties with the composition, such as the superconducting transition temperatures *T*_c_ [103] and magnetic moments (Slater–Pauling curve, see, e.g., [104]), in transition metal alloys composed of neighboring elements. However, as noted 40 years ago [67], this approximation is not good for the properties that depend mainly on *N*(*E*_F_) in alloys composed of elements which are far apart in the periodic table, such as TE and TL elements. In these alloys, the contribution(s) of the d states of the TL(s) to *N*(*E*_F_) is (are) small, if any, as shown in Figure 11, Figure 12 and Figure 13 and demonstrated in numerous literature reports [22,23,24,25,26,95,96,97,98,99]. Therefore, using the full values of the VEC of TLs in calculating the average VEC of an alloy is, apparently, wrong and leads to erroneous variations of the studied properties with such an average VEC. In particular, in crystalline (ScZrNb)_1−x_(RhPd)_x_ alloys with a B2 structure [29], and all TE-TL MGs (e.g., [22,23,24,25,26,67,81,105,106,107,108]), *T*_c_s decreases linearly with increasing average VEC, whereas in disordered films composed of neighboring transition metals from Zr to Tc [103], *T*_c_s increases with increasing VEC over the same range of values of the average VEC. This seemingly unusual behavior has been attributed [29] to the chemical complexity of these quinary alloys, whereas it merely shows that the average VEC does not sufficiently represent *N*(*E*_F_) in alloys having a split band structure of the VB. It was recently shown [22,26] that in all disordered transition metal alloys, regardless of their atomic structure and number of alloying components (and thus chemical complexity), for a given series of transition metals, the *N*(*E*_F_) governs their superconductivity. Note that the impact of chemical complexity is small, at best.

**Figure 14:**
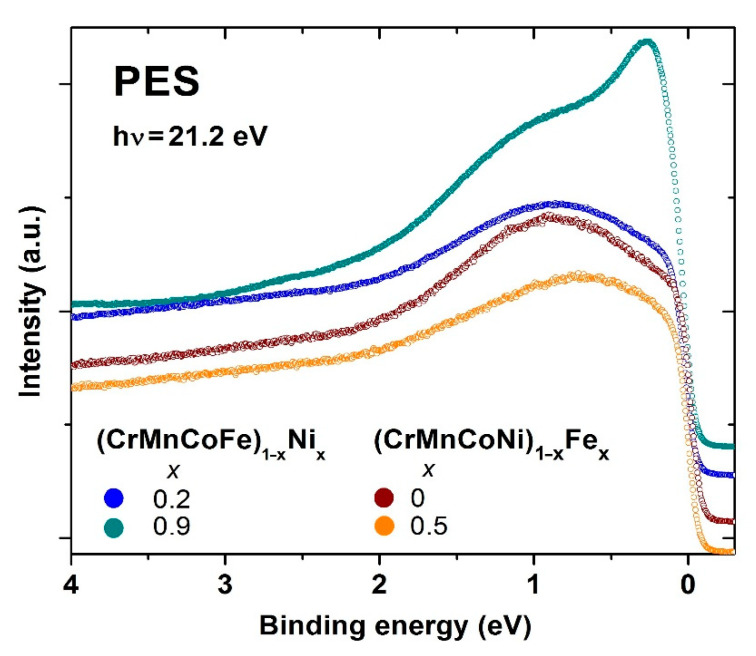
Photoemission spectra of selected (CrMnFeCo)_1−x_Ni_x_ and (CrMnCoNi)_1−x_Fe_x_ alloys. Spectra are displaced upwards for clarity.

Figure 14 shows selected results of the ongoing UPS study of the ES of Cantor-type alloys possessing an FCC crystalline structure. The UPS spectra for (CrMnFeCo)_1−x_Ni_x_ and (CrMnCoNi)_1−x_Fe_x_ alloys, possessing the lowest and highest values of *x*, thus belonging to the HEA and CA concentration ranges, are shown there.

Comparing these spectra with those for the quinary TE-TL MGs in Figure 12, we note that in the Cantor-type alloys, the constituents, being the neighboring elements, seem to form a common band. There are no distinct features in these spectra which are separated in energy, as with those in Figure 11 and Figure 12, denoting the split band structure of the VB. In Figure 14, the prominent feature is a moderate shift in the spectral intensity towards *E*_F_ with increasing *x*. In (CrMnFeCo)_1−x_Ni_x_ alloys, the peak shifts are followed by an intensity increase and narrowing, going from *x* = 0.2 to 0.92, whereas in (CrMnCoNi)_1−x_Fe_x_ a broad peak barely shifts with *x* from 0.85 at *x* = 0 to 0.65 eV at *x* = 0.5, without any significant change of in its shape. In alloys with a variable Ni content, the study of spectra close to *E*_F_ indicated an increase in *N*(*E*_F_) with increasing *x*. Our non-spin-resolved UPS study of polycrystalline samples cannot provide full information about the band structure and magnetic ground state of these alloys. However, in the case of (CrMnFeCo)_1−x_Ni_x_ alloys, a comparison of the measured spectra for selected alloys with these constituent elements (e.g., Ni) and with the results of *ai* calculations of the band structure of some alloys (e.g., [30,40]) provides a qualitative insight into the evolution of the ES with *x*. The spectrum for the alloy with *x* = 0.2 in Figure 14 is qualitatively similar to the calculated DOS of this alloy [30], whereas that of the alloy with *x* = 0.92 is similar to the UPS and XPS spectra of pure Ni. Thus, the ES of these alloys evolves with increasing *x* from the Cantor alloy to that of pure Ni. This insight is strengthened by the results of the simultaneous study of the evolution of magnetism with *x* in the same alloy system, which is described in the next section. In (CrMnCoNi)_1−x_Fe_x_ alloys, the situation is more complex since the shape of spectra hardly changes with x. In addition, the study of spectra close to *E*_F_ does not indicate any systematic tendency of *N*(*E*_F_). The preliminary results for the evolution of magnetism in this system show rather weak magnetic correlations which change rather slowly with the concentration of Fe. Clearly, *ai* calculations are required to obtain better insight into the evolution of the ES and magnetism with the composition in both alloys and their constituents [30]. Very recently, we noted [108] that irrespective of line shapes and peak positions, UPS spectra of all studied alloys can be modeled rather accurately by using three Lorentzian functions (multiplied with Fermi–Dirac distribution functions) with fixed positions of maxima below *E*_F_. The intensities of these maxima are correlated with the concentrations of the constituent elements.

### 3.5. Magnetic and Mechanical Properties

Knowledge of the magnetic state of a system can provide additional information about its ES [44]. Further, the magnetic correlations contribute to the total energy of the system, which can affect its structure and other properties. As noted in the Introduction, in some HEAs and CCAs containing magnetic elements, a novel strengthening mechanism, i.e., magnetic hardening, has been found [18,19]. It is therefore surprising that only a qualitative description of the magnetic state at room temperature [50] was part of detailed studies of the atomic structure and mechanical properties of isopleths made from the components of the Cantor alloy [45,46,47,48]. The exception is the Cantor alloy for which numerous studies, both theoretical and experimental, of its magnetism exist (e.g., [30,40,41,42,43,104,109,110,111]). However, the descriptions of the magnetism of this alloy obtained in different studies differ considerably as regards the Curie temperature *T*_C_ and the average magnetic moment per atom (or formula unit) *m*. This may not be surprising considering the chemical complexity of the equiatomic CrMnFeCoNi alloy, composed of five magnetic elements, each one with a concentration just above the site percolation threshold on an FCC lattice. Accordingly, there exists a wide distribution of atomic environments within the alloy which results in a very wide distribution of the exchange interactions between the constituent atoms, and a similarly wide distribution of the values and directions of the magnetic moment for each constituent atom [19]. In theoretical studies, which invariably predict the ferromagnetic ground state of the Cantor alloy, the results for *T*_C_ and *m* depend on the method of the employed calculation [42], e.g., *T*_C_ = 23 K and *m* = 0.39 *μ*_B_ were calculated in [30]. Almost all experimental studies also find a ferromagnetic ground state in this alloy but report very different results for the corresponding parameters *T*_C_ and *m*. This observed scatter of measured *T*_C_ and *m* values was most probably caused by the difficulty to obtain a homogeneous distribution of elements in the solid solution (as illustrated in Supplementary Figures S1 and S2) due to the different bonding tendencies between different constituents (inset in Figure 3), as discussed in some detail elsewhere [45,46,47,48]. One particular study [104] attributed low values of *T*_C_ and *m* of the Cantor alloy to its anti-invar behavior, similar to that of the high-temperature γ-Fe phase which has an FCC structure and a lattice parameter close to that of the Cantor alloy. Accordingly, substantial enhancement of the magnetic parameters of the Cantor alloy in respect to undoped alloys was obtained by expanding its lattice by doping it with carbon. However, the Cantor alloy with the addition of Cr and Mn contributed to the weak, if any, magnetism since the equiatomic FeCoNi alloy with an FCC structure has a *T*_C_ close to 1000 K [112].

**Figure 15:**
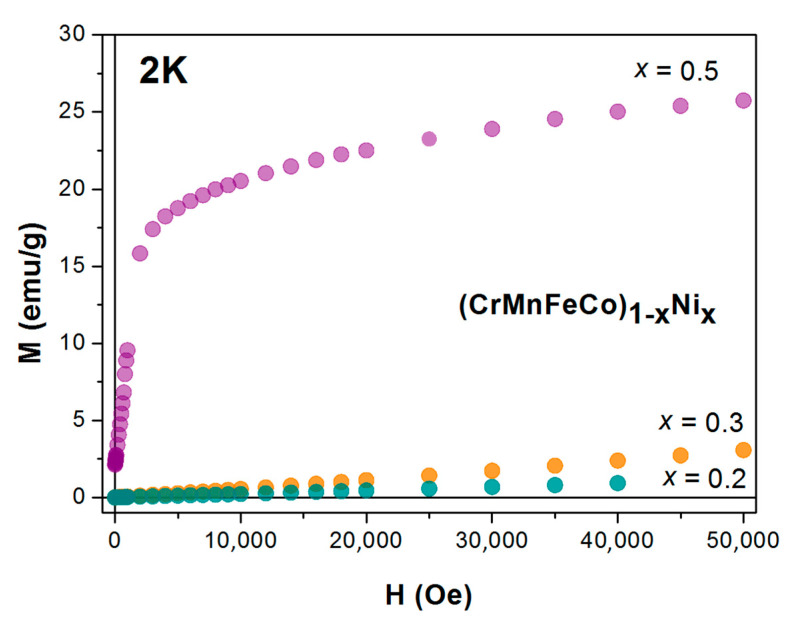
Magnetization isotherms of (TiZrNbCu)_1-x_Ni_x_ MGs with the lowest Ni content measured at a temperature of 2 K.

**Figure 16:**
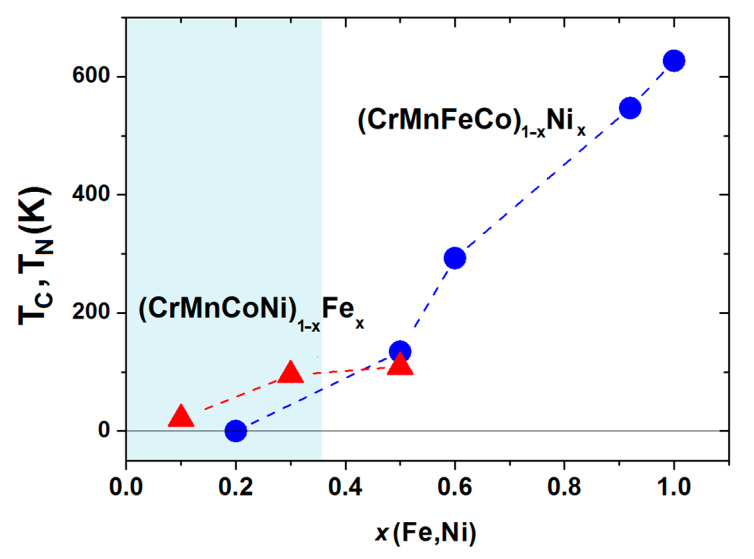
Magnetic phase diagrams of (CrMnFeCo)_1−x_Ni_x_ and (CrMnCoNi)_1−x_Fe_x_ alloys. Dashed lines are a guide for the eyes. *T*_C_ and *T*_N_ denote the Curie (circles) and peak/Neel (triangles) temperatures, respectively.

In Figure 15 and Figure 16, the first results of the ongoing study of the evolution of the magnetism in (CrMnFeCo)_1−x_Ni_x_ (*x* = 0.2, 0.3, 0.5, 0.6, 0.92 and 1.0) and (CrMnCoNi)_1−x_Fe_x_ (*x* = 0, 0.1, 0.3 and 0.5) alloys with a single-phase FCC structure are presented. Figure 15 shows the M–H data measured at the temperature of 2 K for the alloys with low Ni contents. The linear M–H variation for *x* = 0.2 shows that the ground state of the Cantor alloy with a rather homogeneous distribution of constituent elements is nonmagnetic. Thus, the ferromagnetism observed in numerous experiments is probably associated with the presence of magnetic clusters in an insufficiently homogenized sample. On the other hand, the ferromagnetism predicted in theoretical calculations is probably caused by the intricacy of these calculations. We note that *T*_C_ = 10 ± 10 K was predicted by Monte Carlo simulation of the Cantor alloy, indicating, in fact, a nonmagnetic state of this alloy [42].

The magnetic susceptibility of the Cantor alloy in Figure 15 has a similar value to that of the Hf_1-x_Fe_x_ MGs [113] with *x* around 0.4, which correspond to a superparamagnetic state. Although the M–H curve for the alloy with *x* = 0.3 is qualitatively similar to that of the Cantor alloy (Figure 15), it shows a somewhat more complex magnetic behavior at low temperatures. A detailed study of the M–H loops of this alloy in the temperature range 5–50 K revealed a very small hysteresis curve superposed on a large linear superparamagnetic term at temperatures below about 35 K. The very small *m* = 0.008 *μ*_B_ corresponding to this ferromagnetic contribution at 5 K indicates the presence of magnetic clusters with a low *T*_C_, within the superparamagnetic host, rather than bulk ferromagnetism. The origin of the formation of clusters within this alloy is not yet clear (the insufficient homogenization (see Supplementary Materials Figure S1), or the vicinity of the magnetic percolation threshold). The M–H curve of the alloy with *x* = 0.5 is ferromagnetic, but a detailed study of its transition to a paramagnetic state above *T*_C_, using the ac susceptibility, revealed a strong magnetic inhomogeneity, similar to that observed in Hf-Fe and Zr-Fe MGs with Fe contents above the ferromagnetic percolation threshold. The magnetic phase diagram of (CrMnFeCo)_1-x_Ni_x_ alloys is shown in Figure 16. For *x* ≥ 0.5, the values of *T*_C_ monotonically increase with *x* and smoothly join those of pure Ni at *x* = 1. This is in accordance with the results of UPS for the same Cantor alloys which show the evolution of the DOS for *x*, ranging from the Cantor alloy (calculated DOS only [30,40]) to the alloy close to pure Ni (*x* = 0.92), as illustrated in Figure 14. The preliminary results for the low-temperature magnetic moments of the alloys with *x*≥0.5 indicate a monotonic increase in *m* with the Ni content to that of pure Ni, *m* = 0.62*μ*_B_, at *x* = 1, qualitatively similar to that of *T*_C_ in Figure 16. Therefore, the deviations of the average atomic volumes from Vegard´s law and of the hardness from the Mott–Nabarro–Labush law observed in the HEA composition range [45] coincide with the disappearance of a long-range magnetic order and with the formation of magnetic inhomogeneities on an atomic scale. Thus, electronic correlations seem to favor both HEA formation and the disappearance of magnetism in the HEA range. Simultaneously, the onset of a ferromagnetic order strongly enhances the thermal stability of the alloys in the CA range, as shown in Figure 5.

The preliminary results for the characteristic temperatures of (CrMnCoNi)_1−x_Fe_x_ alloys are also shown in Figure 16. The sharp cusp in the magnetization and ac susceptibility of alloys with *x* ≥ 0.3, accompanied by relatively low values of magnetization and magnetic susceptibility, indicates antiferromagnetic transitions occurring at the Neel temperatures *T*_N_ of around 100 K, which is close to that of γ-Fe. Thus, these alloys may, as with the Cantor alloy [104], show anti-invar behavior (an anomalous thermal expansion at elevated temperatures). At present, it is not clear to us the true origin of the relatively broad peak in the ac susceptibility of the alloy with *x* = 0.1 situated around *T*_P_ = 21 K. Notice that (CrMnCoNi)_1−x_Fe_x_ alloys are distinguished from other isopleths studied at ICMPE [45,46,47,48] in that their VEC = 8 does not depend on *x*. Thus, the appearance of a phase with a BCC crystalline structure for *x* > 0.5 [47] probably indicates the limitations of a simple VEC criterion for the selection of the crystalline phase in CCAs.

A rather detailed discussion of magnetic susceptibilities of three quinary TE-TL MG systems has recently been reported [24,25,52]. In this paper, we will address the compositional variations of the room temperature magnetic susceptibilities, *χ*_exp_, of these alloy systems. Due to the close relationship between the magnetic susceptibility and ES [44], the corresponding results of UPS and LTSH studies will be used to explain the differences in the *χ*_exp_ behavior in the three alloy systems.

**Figure 17:**
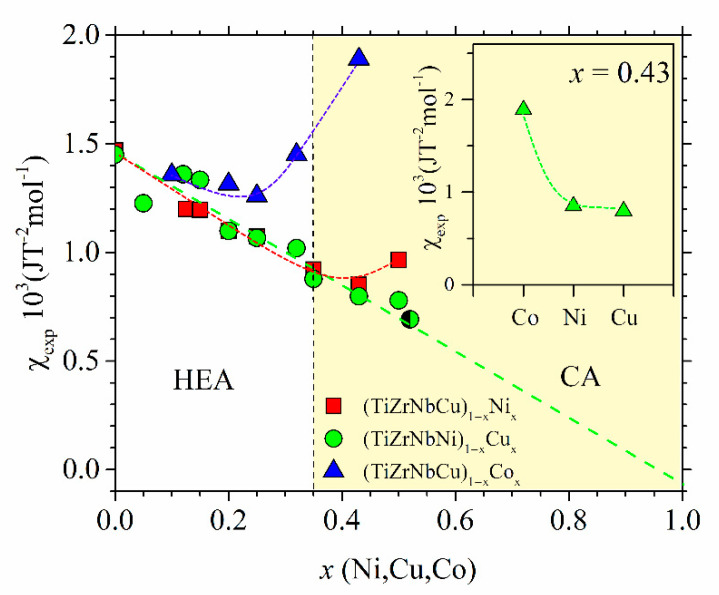
Room temperature magnetic susceptibility, *χ*_exp_, of (TiZrNbNi)_1−x_Cu_x_, (TiZrNbCu)_1−x_Ni_x_ and (TiZrNbNi)_1−x_Co_x_ MGs vs. *x*. The inset: *χ*_exp_ of MGs with *x* = 0.43 Co, Ni and Cu.

The variations of χ_exp_ with the TL content for all our alloys are shown in Figure 17. These variations are qualitatively similar to those observed in the corresponding binary Zr_1−x_TL_x_ MGs with TL = Cu, Ni or Co [66,67,81,95,105,107,108]. The data for all alloy systems in Figure 17 for *x* = 0 appear to extrapolate to the same value, that of pure amorphous Zr, equal to 1.5 10^−3^ JT^−2^mol^−1^ [81,114]. In addition to comparable initial values, the data for all alloy systems in Figure 17 also show similar initial variations with *x*: the small initial decrease on increasing *x* to *x* ≤ 0.2 probably shows that the d states of TEs dominate both χ_exp_ and *N*(*E*_F_) at low contents of TL (Figure 11, Figure 12 and Figure 13). For *x* > 0.2, two distinctly different types of behavior of χ_exp_ can be seen in Figure 17. The χ_exp_ of (TiZrNbNi)_1−x_Cu_x_ MGs linearly decreases throughout the explored concentration range, *x* ≤ 0.52, and extrapolates close to that of pure Cu for *x* = 1 [25], whereas these for (TiZrNbCu)_1−x_Ni_x_ and (TiZrNbCu)_1−x_Co_x_ pass through a minimum value at some system-dependent *x* and then start to increase at higher concentrations.

The behavior of χ_exp_ of (TiZrNbNi)_1−x_Cux MGs is qualitatively the same as that of *γ* (Figure 13) and *N*(*E*), which is characteristic of the ideal solution [25]. The same behavior of both *χ*_exp_ and *N*(*E*) has been previously observed in all binary TE_1−x_Cu_x_ MGs with TE = Ti, Zr or Hf [66]. In all these systems, the atomic parameters, such as the average atomic volumes, vary linearly with the concentration according to Vegard’s law (Figure 1), whereas their APFs and coordination numbers, *N*, are constant, independent of *x* [25,66,95]. The behavior of *χ*_exp_ of (TiZrNbCu)_1−x_Ni_x_ is also qualitatively the same as that of *γ* and *N*(*E*_F_) [24,26]. All these quantities exhibit an increase at the same value of *x* ≥ 0.43. Qualitatively, the same correlation between the variations of *χ*_exp_ and *γ* and *N*(*E*_F_) are observed in binary Zr_1−x_Ni_x_ MGs, with the distinction that the increase in all these quantities sets off around the concentration corresponding to the band crossing in this system, *x* = 0.67 [24,74].

The crossover of *χ*_exp_ and *γ* in the studied alloys takes place at significantly lower values of x compared to binary MGs [24,74], which is surprising as the Hall effect measurements [26] showed the band crossing in (TiZrNbCu)_1−x_Ni_x_ alloys at a higher concentration than that of the corresponding Zr_1−x_Ni_x_ MGs [74]. An enhanced electronic correlation favoring magnetic ordering (such as the Stoner enhancement [24,25,52]) in respect to that in Zr_1−x_Ni_x_ MGs [105] is a possible explanation of the increases in both *χ*_exp_ and *γ* in the studied system at a concentration considerably below that expected for band crossing. Alternatively, a change in local atomic arrangements visible as a sudden increase in *N* at *x* ≥ 0.43 [24] may be responsible for the simultaneous increases in *χ*_exp_ and *γ* in (TiZrNbCu)_1−x_Ni_x_ alloys. In the (TiZrNbCu)_1−x_Co_x_ alloys in Figure 17, as in (TiZrNbCu)_1−x_Ni_x_ alloys [24], the increase in *χ*_exp_ at *x* ≥ 0.25 coincides with that in the APF (Figure 10) and *N* [62], and thus with some change in local atomic arrangements.

Further, this *χ*_exp_ increase occurs at the concentration of Co which is much lower than that estimated for the band crossing, *x* = 0.68 [52]. In the corresponding binary Zr_1−x_Co_x_ MGs, this *χ*_exp_ increase appears for *x* > 0.4. At the same value of *x*, which is close to the concentration at which the band crossing occurs in these MGs, *x* = 0.5 [74,75], *N*(*E*_F_) also starts to increase with increasing *x* [108].

Unfortunately, there are only two experimentally determined values for *γ* in (TiZrNbCu)_1−x_Co_x_ alloys (*γ* = 4.1 and 4.7 mJ/mole K^2^ for *x* = 0.2 and 0.43, respectively, Figure 13 and Reference [101]), preventing any detailed comparison between the variations of *γ* and of *χ*_exp_ with the Co content. However, recent magnetization measurements of our partially crystalline alloy with *x* = 0.5 showed very small ferromagnetic hysteresis loops at low temperatures, *T* = 2 K and 30 K. Thus, the threshold for the onset of ferromagnetism in our alloys seems to be lower than that in binary Zr_1−x_Co_x_ MGs, which is around *x* = 0.6 [105,108]. Accordingly, the Stoner enhancement (S) should increase more rapidly in the studied quinary alloys than that in similar binary MGs which, in turn, may explain the observed enhancement of *χ*_exp_ at a relatively low Co content (within the HEA concentration range, Figure 17).

In conclusion, we notice the self-consistency between the measured ES, local atomic arrangements and magnetic susceptibility in the studied quinary TE-TL MGs. The inset of Figure 17 illustrates an intimate relationship between the ES, represented by γ, and intrinsic properties (such as the magnetic or mechanical properties) of the same alloys, addressed in the previous section in the discussion of Figure 13. The data in the inset of Figure 17 show the values of *χ*_exp_ for the alloys with *x* = 0.43 of Co, Ni or Cu. The variation of *χ*_exp_ is almost the same as that of *γ* shown in the inset of Figure 13. This demonstrates that a change in *χ*_exp_ on crossing from the HEA to the CA concentration range is largely due to the corresponding ES (more precisely, the degree of the presence of d states of TLs at *E*_F_, and thus the amount of band splitting, Figure 12). Thus, knowledge of the evolution of the ES with the composition is perhaps the simplest way of predicting the change in intrinsic properties on the transition from the HEA to the CA concentration range. However, as discussed elsewhere [24,25,26,32,33,52,66], the striking similarity between the variations of *χ*_exp_ and *γ* or *N*(*E*_F_) (cf., Figure 13 and Figure 17) is quite surprising considering the complexity of the *χ*_exp_ in transition metals and their alloys and the fact that only one term out of three major ones is a function of *N*(*E*_F_) [66,100].

Several outstanding mechanical properties such as a very large strength (approaching the theoretical strength) as well as a very high hardness, fracture toughness and wear resistance make metallic glasses interesting for diverse practical applications [89,115]. All these properties of MGs are strongly affected by their disordered atomic structure and macroscopic homogeneity. The very large hardness of MGs compared to that of crystalline alloys is associated with the absence of extended crystal defects (caused by structural disorder) which results in the elastic-plastic type of deformation. Hence, the yield stress is practically the same as the tensile limit. Thus, the hardness of MGs is proportional to both the yield stress and Young’s modulus *E* [116]. Since MGs are macroscopically homogenous and isotropic, their hardness is also proportional to their shear and bulk modules, *G* and *B*, respectively. All these properties are directly related to the strength of interatomic bonding [22,23,24,25,37,66,81,82,83,84] as a consequence of the absence of the extended crystal defects and associated crystal slip mechanisms. Since interatomic bonding strength also affects the average atomic volume, the thermal parameters such as *T*_m_, *T*_l_ and *T*_x_ and atomic vibrations are consequently also correlated with the mechanical properties of MGs [24,25,37,81,82,83,84,89,117]. Considering a wealth of information that can be obtained from rather simple hardness measurements in MGs, it seems surprising that the first systematic study of the variation of microhardness, *H*_V_, with the composition, covering both HEA and CA concentration ranges, has been performed only recently on (TiZrNbCu)_1−x_Co_x_ MGs [52]. This study has shown that the correlation between *H*_V_ and some other parameters associated with interatomic bonding and the ES, well established for binary and ternary MGs, also applies to quinary ones. Moreover, it has been shown that by averaging enough results, the variation of *H*_V_ can reflect a subtle change in local atomic arrangements such as that affecting the APF in Figure 10. Below, we provide new results for the *H*_V_ of (TiZrNbCu)_1−x_Cu_x_ and (TiZrNbCu)_1−x_Ni_x_ MGs and compare the results of *H*_V_ with those for some other properties of the same alloys, as well as with results for hardness obtained from nanoindentation, *H*_nano_, in selected Cantor-type alloys with an FCC crystalline structure [45,47,48].

**Figure 18:**
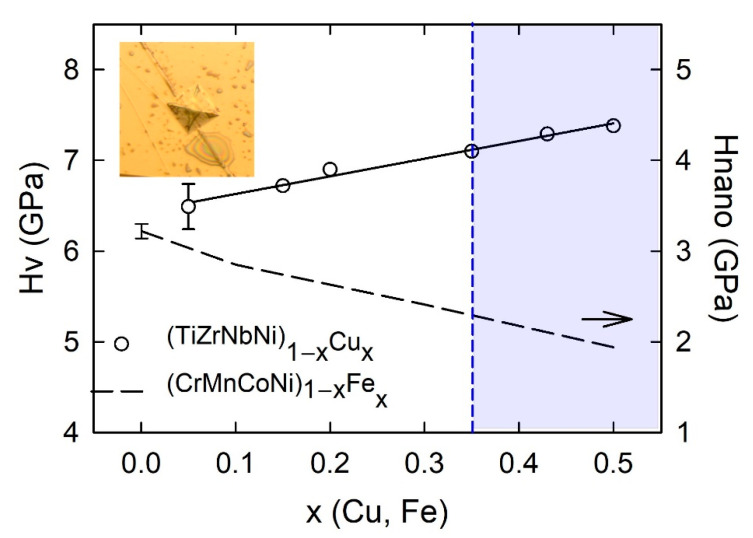
Room temperature Vickers microhardness, *H*_V_, of (TiZrNbNi)_1−x_Cu_x_ MGs and nanohardness, *H*_nano_, (dashed line) of (CrMnCoNi)_1−x_Fe_x_ alloys vs. *x*. The inset: the image of a typical indentation on the surface of an MG sample.

Variation of the room temperature Vickers microhardness of our as-cast (TiZrNbCu)_1−x_Cu_x_ with the Cu content on crossing from the HEA to the CA concentration range is shown in Figure 18. The inset shows an image of a typical indentation showing its well-defined edges. The error bar on *H*v for *x* = 0.12 denotes the standard deviation which was around 5% for all compositions.

**Figure 19:**
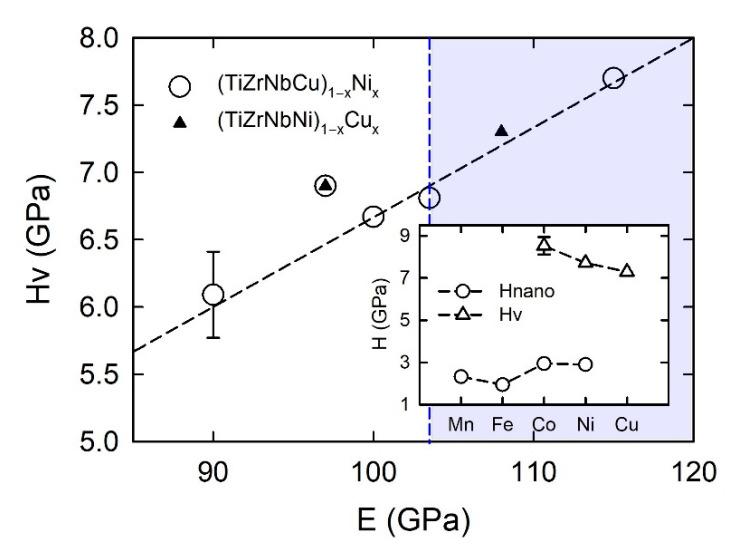
A plot of *H*_V_ vs. *E* for (TiZrNbNi)_1−x_Cu_x_ and (TiZrNbCu)_1−x_Ni_x_ MGs. The inset: *H*_V_ of MGs with *x* = 0.43 and *H*_nano_ for Cantor-type alloys with *x* = 0.5 Mn, Fe, Co and Ni.

A linear increase in *H*_V_ with *x* is consistent with the corresponding increase in the interatomic bonding showing up in an increase in thermal parameters such as *T*_x_ and *T*_l_ (see Figure 4 of [25]). Further, the *H*_V_ (*x*) variation is apparently correlated with the variations of the average atomic volume and the mass density of the same alloys (Figure 6 in [25]). Indeed, it seems plausible that in alloys with a similar atomic structure (amorphous), hardness increases with density. The increase in *H*_V_ with increasing *x* is accompanied by a linear decrease in *γ* and *N*(*E*_F_), as shown in Figure 13. Thus, the variation of *H*_V_ with the composition in these MGs (showing no change in variation on crossing from the HEA to the CA concentration range, Figure 18) is consistent with their ideal solution behavior [25]. Further, it qualitatively shows the same correlation with the corresponding ES and with parameters associated with interatomic bonding as those previously observed in binary and ternary TE-TL MGs [65,81,82,83,84,89,117]. As seen in Figure 18 and explained in the previous paragraph [82,89,115], the magnitude of *H*_V_ is very large and increases from about 6 to 7.3 GPa for *x* = 0 and 0.5, respectively. It is of interest to compare these values with those of (TiZrNbCu)_1−x_Co_x_ [52] and (TiZrNbCu)_1−x_Ni_x_ MGs and with those for similar binary TE-TL MGs [81,82,83,84]. As seen in the inset of Figure 19, in quinary TE-TL MGs at *x* = 0.43, *H*_V_ decreases monotonically, going from TL = Co to Cu. This variation is similar to the variation of γ in the inset of Figure 13 and that of *χ*_exp_ in the inset of Figure 17 (both taken at the same *x* = 0.43) which shows that the variation of *H*_V_ is similar to that of γ and *χ*_exp_ determined with the contribution of the d states of the given TL to *N*(*E*_F_). We note that such a rather simple correlation between *H*_V_ and *N*(*E*_F_) is believed to be specific to TE-TL MGs and uncommon in crystalline alloys [81,82,83,84]. However, a recent study of the crystalline structures and mechanical properties of (TiZrHf)_x_(CuNi)_1−x_ crystalline alloys with 0.4 ≤ *x* ≤ 0.8 showed that the yield strength decreases with increasing *x* (and thus the TE content) even though the crystalline phases also change with the concentration [51]. This probably implies that the effect of the ES on the strength of quinary TE-TL crystalline alloys is stronger than that of the corresponding crystalline phase(s). Finally, we compared the value of *H*_V_ of the alloy with *x* = 0.5 in Figure 18 with that of equiatomic binary Ti-Ni or Cu (*H*_V_ around 7 GPa) and Zr-Ni or Cu (*H*_V_ around 5 GPa) MGs to deduce the effect of chemical complexity on the hardness of TE-TL MGs. This comparison shows that the type of TE constituent (such as Ti or Nb) is probably more important for the magnitude of *H*_V_ than the number of alloying components.

The dashed line in Figure 18 illustrates the variation of *H*_nano_ of single-phase (CrMnCoNi)_1−x_Fe_x_ FCC alloys with *x* [47]. As expected, for crystalline alloys, due to crystal slip mechanisms, the values of *H*_nano_ are a factor of two to four times smaller than the values of *H*_V_ of (TiZrNbCu)_1−x_Cu_x_ MGs. *H*_nano_ decreases approximately linearly with *x* and extrapolates around 0.8 GPa for pure FCC iron, *x* = 1. The observed variation was reproduced quite well [47] by using the model for solid solution strengthening in FCC HEAs. The inputs in this model [118] are the atomic volumes of the alloying components and the corresponding elastic constants (the shear modulus and the Poison ratio). The predictions of the model are more sensitive to the values of atomic volumes than those for elastic constants. The linear decrease in the atomic size mismatch *δ* of these alloys with *x* shown in Figure 3 seems consistent with the observed variation of *H*_nano_. We note that only in (CrMnCoNi)_1−x_Fe_x_ alloys did *H*_nano_ decrease on increasing *x.* In alloy systems with a variable content of Co or Ni, it increased with increasing *x* within the same concentration range. This is illustrated in the inset of Figure 19 which shows the values of *H*_nano_ for *x* = 0.5 of Co, Mn, Fe and Ni. These values show a deep minimum at Fe. The error bar on *H*_nano_ for *x* = 0 denotes the standard deviation, which was around 5% for all compositions.

Two other isopleths of Cantor alloys that can be formed in a single-phase FCC structure, for Mn or Ni contents up to *x* ≥ 0.5, showed more diverse compositional variations of the studied properties [47]. In the isopleth with a variable Mn content, (CrFeCoNi)_1−x_Mn_x_, a strong linear increase in the average atomic volume on increasing *x* is accompanied by a small and shallow maximum of hardness centered around *x* = 0.1 (thus within the HEA range, as illustrated in Figure 1). This maximum was described reasonably well [47] using the model for strengthening of random FCC alloys [118]. Since in this system, the melting temperatures calculated by the Calphad method decrease rapidly with increasing Mn content [46], it seems plausible that a combination of solid solution strengthening and decreasing the bonding strength on increasing *x* affects the observed change in hardness and may explain the occurrence of the maximum at a low *x*. The hardness of the isopleth with a variable Ni content showed a quite large and broad maximum centered around *x* = 0.6 Ni (thus within the CA concentration range, as illustrated in Figure 1) accompanied by a similarly complex variation of the average atomic volume: a linear decrease according to Vegard’s law for *x* ≥ 0.6 and a strong negative deviation from Vegard’s law at a lower *x* [45]. The magnetic phase diagram of the same alloys presented in Figure 16 shows that at *x* = 0.6, a long-range ferromagnetic order persists up to above room temperature. Therefore, the deviations of the average atomic volumes from Vegard´s law and those of hardness from the Mott–Nabarro–Labush law [45] coincide with the disappearance of the long-range magnetic order and with the formation of magnetic inhomogeneities on an atomic scale in the HEA concentration range. The magnitude and variation of melting temperatures within the HEA/nonmagnetic concentration range in Figure 5 indicate a significantly reduced strength of interatomic bonding in this regime, compared to that predicted by the rule of mixtures.

In Figure 19, we show the result of the experimental check of the validity of the universal relationship between *E* and *H*_V_ of metal–metal-type (M–M) binary and ternary MGs [116], where *E* = 15 *H*_V_ in our quinary TE-TL MGs. The values of *H*_V_ of all (TiZrNbCu)_1−x_Cu_x_ and (TiZrNbCu)_1−x_Ni_x_ MGs for which the corresponding values of *E* exist are plotted in an *H*_V_ vs. *E* diagram in Figure 19. Considering the fairly large errors inherent in measurements of microhardness, the data agree quite well with the universal relationship between *E* and *H*_V_ of metal–metal-type (M–M) binary and ternary MGs denoted with the dashed line [115]. Finally, we used this relationship to calculate the value of *E* for pure amorphous Cu by multiplying the value of *H*_V_, obtained by extrapolation of the values in Figure 18 to *x* = 1, by 15. The resulting *E* = 120 GPa agrees well with that of pure FCC Cu. The same relationship also provided a good estimate of *E* of pure amorphous Co [52].

Distinct features of MG such as a disordered atomic structure, macroscopic homogeneity and the absence of extended crystal defects possessing several remarkable mechanical properties are also favorable for some other functional properties of these materials. For example, the exceptional resistance to radiation and corrosion of many MGs is associated with their disordered atomic structure and the absence of extended defects [89]. Moreover, Zr-based BMGs are known to possess exceptional irradiation resistance [119] and corrosion resistance properties [120], while some a-HEAs/CCAs containing rare-earth elements [58,121] have promising properties of magnetic refrigerants due to effects of the disordered atomic structure and compositional tuning of the magnetic ordering temperature.

## 4. Conclusions and Outlook

In this work, we have presented an overview of two comprehensive studies of the transition from the regime of high-entropy alloys (HEAs) to that of conventional alloys (CAs) with the same chemical make-up performed in two very different types of alloy systems: isopleths of Cantor-type alloys with a single-phase FCC crystalline structure [45,46,47,48] (first group of HEAs in [13]) and in a type of nonlinear alloy [6], quinary metallic glasses (MG) composed of early (TE) and late transition metals (TL) [22,23,24,25,26,37,52,62]. Altogether, all five isopleths of the Cantor-type alloys and three systems of quinary TE-TL MGs composed of a total of nine elements (Ti, Cr, Mn, Fe, Co, Ni, Cu, Zr and Nb) combined in around fifty different alloy compositions were the focus of our experimental research. The overview of some of the published results for these alloys is complemented with numerous novel results, and the already reported results are complemented with new ones and/or analyzed in a different manner.

The properties discussed include those associated with the (i) electronic structure, studied both by photoemission spectroscopy and by low-temperature specific heat measurements, (ii) parameters related to the atomic structure and local atomic arrangements studied by X-ray diffraction, (iii) thermophysical parameters associated with the atomic size mismatch and the strength of interatomic bonding, (iv) the thermal stability and the glass-forming ability, GFA (of MG-forming alloys), (v) the magnetic properties (including magnetization, magnetic susceptibility and the temperatures of magnetic transitions) and (vi) a few mechanical properties, mostly hardness and some data for Young´s modulus. When appropriate, the observed properties and their compositional variations were compared with those for similar alloy systems, such as quinary crystalline TE-TL alloys [29,51] and binary and ternary TE-TL MGs [22,23,24,25,52,66]. Some properties and their variations with the composition, especially the thermal stability parameters and lattice parameters, were compared with those calculated by using the rule of mixtures, i.e., Vegard´s law.

These mostly intrinsic properties were found to exhibit variations with the composition of alloying components, depending on the studied alloy and selected property, as illustrated in Figure 1. The type of observed variation and the composition for which the selected property shows the largest value (*P*_b_) depend on the evolution of the ES and the related atomic structure, i.e., on the evolution of interatomic interactions and electronic correlations with the composition in a particular system. This is nicely demonstrated in systems exhibiting linear variations of the explored properties (denoted with VL in Figure 1), such as the (TiZrNbNi)_1−x_Cu_x_ MGs exhibiting the ideal solution behavior [25] and (CrMnCoNi)_1−x_Fe_x_ crystalline alloys with an FCC lattice.

It is remarkable that both of these alloy systems with very different atomic structures and shapes of the DOS, in other words, different ESs, show simple linear variations of the explored properties. However, in (TiZrNbNi)_1−x_Cu_x_ MGs, the decrease in the lattice parameter and average atomic volume with increasing *x* is accompanied by an increase in the hardness due to an increase in the bonding strength. Conversely, in single-phase (CrMnCoNi)_1−x_Fe_x_ crystalline alloys, both the lattice parameter and the hardness decrease with increasing *x*. As noted in the discussion of the room temperature Vickers microhardness results, the strong, linear decrease in H_V_ on increasing the Fe content is accompanied by a similar decrease in the atomic size mismatch, which is described quite well with the model for solution strengthening of random FCC alloys [47,118]. The observed decrease in hardness may also indicate a decreasing stability of the FCC phase on increasing iron content. The strong, linear decrease in atomic volumes of (CrMnFeNi)_1−x_Co_x_ alloys with increasing *x* was accompanied by a fairly strong increase in hardness, which was also described rather well with the same model [118]. Notice that, in CCAs with an FCC structure composed of 3d metals, the increase in the Co content seems to invariably increase their strength [50], whereas that of Fe seems to decrease it. The compositional variations of the properties of isopleths with a variable Mn or Ni content are more diverse [47]. In these isopleths, hardness showed a maximum in the HEA region for a variable Mn content and in the CA region for a variable Ni content. Thus, variations of hardness in these four isopleths followed all compositional variations depicted in Figure 1. Further, a simple change in one main principal component with another produces a drastic change in the compositional variation of the studied property in Cantor alloys.

As discussed in some detail throughout this paper, the possible change in the variation of the properties of the studied quinary TE-TL MGs is correlated with the split band structure of their DOS, particularly with the position of the 3d states of the TL in respect to the Fermi level, *E*_F_, of the alloy. Accordingly, the properties of (TiZrNbCu)_1−x_Co_x_ MGs change their functional dependence with the composition within the HEA concentration range. Those of MGs with a variable Ni content change in the CA regime, whereas in MGs with a variable Cu content, showing an ideal solution behavior, the change in the functional dependence probably does not take place for *x* < 1. Since the split band structure of the DOS is generic to TE-TL MGs, qualitatively, the same dependence of the change in a compositional variation on a selected TL was previously observed in binary and ternary TE-TL MGs (e.g., [74]). Although such a simple correlation between the position of the TL element in the periodic table with the change in properties with the composition in the corresponding TE-TL MGs may be enhanced by their amorphous atomic structure, it probably also exists in all crystalline TE-TL alloys. This observation was supported by a similar variation of the yield strength in (TiZrHf)_x_(NiCu)_1−x_ crystalline alloys [51] to that of the Vickers hardness, *H*_V_, of (TiZrNbNi)_1−x_Cu_x_ MGs. In the same way, support for this simple correlation comes from the qualitatively similar variation of superconducting transition temperatures in crystalline quinary TE-TL alloys with a B2 (BCC) structure with the TL content [29] to that in our quinary and numerous binary and ternary TE-TL MGs [26,67].

Two additional important results obtained for the studied quinary TE-TL MGs are also likely to be generally valid in multicomponent MGs. The first study of the crystallization temperatures and enthalpies for the first and last crystallization events revealed that both the magnitudes and variations with the composition of these parameters are different for different crystallization events. This result supports a previous claim [52] that one reason for the discrepancy between the rather good thermal stability of high-entropy MGs (HE-MG) and their modest GFA has an origin in the fact that these properties are associated with different crystallization events. Simultaneously, the primary crystallization of a metastable BCC phase in a TiZrNbCuNi MG with a lower Ni content provided a plausible explanation for the rather low corresponding crystallization enthalpy. The experimental proof that a simple relationship between Young´s modulus and microhardness, generally valid for binary MGs of the metal–metal type, is also applicable to HE-MGs and other multicomponent MGs makes the research of *H*_V_ of these MGs very important; simple measurement of microhardness provides a good insight into both elastic modules and the yield strength [116].

The documented possibility to tune the properties of both the quinary TE-TL MGs and Cantor-type crystalline alloys by changing the composition and/or the selected principal alloying constituent (e.g., Mn, Fe, Co, Ni or Cu) may be useful for the fabrication of alloys and/or coatings with predetermined properties and their compositional variations (Figure 1). This emphasizes the importance of studying the transition from HEAs to conventional alloys with the same chemical make-up. Before we look at additional research results that can provide a deeper understanding of the alloys studied, we will briefly summarize some key findings of our previous research:

(i) HEA properties are not necessarily superior to their lower configuration entropy derivatives;
(ii) The ES determines the compositional variation of the intrinsic properties in all these alloys;
(iii) The composition with the best properties in a given alloy system depends on the selected property (e.g., magnetic or mechanical);
(iv) *ai* calculations accompanied by experimental ES research and magnetic properties can provide deeper insight into the transition from an HEA to a CA in the studied system (and hence answer the question: which is better?).

Although a satisfactory description has been provided for almost all results obtained from the studies of both quinary TE-TL MGs and isopleths based on the Cantor alloy, some additional research of these systems may provide still a deeper insight into their properties and compositional effects. For isopleths based on the Cantor alloy, it would be worth reducing the gap between the concentration range of the FCC phase predicted by Calphad calculations [46] and that observed [47] (in the case of alloys with variable Fe, Co and possibly Mn contents) using melt spinning and, eventually, subsequent homogenization annealing. If this approach proves realistic, it would provide a better insight into the evolution of the electronic structure, magnetic properties and elastic properties of these systems. There is no doubt that experimental determination of the elastic modules in all five isopleths would be better than their calculation using the rule of mixtures [47]. These experimental elastic modules, such as *T*_m_, can provide an independent insight into the compositional variation of the bonding strength in these alloys. The good consistency between the results for the ES and magnetic and mechanical properties observed in alloys with variable Ni contents calls for continued research into the ES and magnetic properties of other Cantor alloy isopleths. The extension of studies of the transition from HEAs to CAs to ternary (e.g., [50]) and/or quaternary CCAs of 3d metals would provide deeper insight into the effects (and importance) of the chemical complexity in this type of alloy.

For the studied TE-TL MGs, systematic study of the crystallization events and their products would be interesting. Comparison of these results with those obtained from a parallel study of Ti-Zr-Hf-Cu-Ni alloys (which form bulk metallic glasses [1]) could be important for understanding the GFA in a-HEAs. Even more important, both conceptually and for possible applications, would be the study of multicomponent alloys that can be fabricated in both ordered crystalline phases and amorphous phases [51,54]. By adjustment of their compositions and appropriate processing, these alloys may exhibit outstanding mechanical properties. They may also help to disentangle the effects of disorder in multicomponent alloys, which is an important conceptual problem. Some other problems of fundamental interest that can be effectively studied using HEAs/CCAs (such as the metal–insulator transitions recently observed in a new class of 2D HEAs [122]) have been mentioned in the Introduction and in previous publications [22,24].

With the aspiration to encourage further efforts in finding the optimal alloy composition with desirable properties, our belief is that the most reliable way to do this would be by conducting experimental measurements of magnetic properties and valence band properties by photoelectron spectroscopy combined with *ai* calculations.

## Data Availability

Reasonable requests for data can be addressed to M.L.-B., A.S.F., I.A.F., R.R., K.Z., P.P., V.M.T. and Đ.D.

## Supplementary Materials

The following are available online at www.mdpi.com/article/10.3390/ma14195824/s1, Figure S1: SEM-BSE image of a surface of a (CrMnFeCo)_0.7_Ni_0.3_ alloy with corresponding elemental mappings of (**a**) as-cast and (**b**) sample annealed at 1373 K for 6 h. The dendritic structure in (**a**) with marked difference in composition (2–5 at. %) between the dendritic (rich in Mn) and interdendritic (some excess of Fe and Co) regions in (**a**) disappears upon annealing in (**b**). Figure S2: SEM-BSE image of a surface of a (CrMnFeCo)_0.92_Ni_0.08_ alloy and corresponding elemental mappings for (**a**) as-cast and (**b**) sample annealed at 1373 K for 6 h. The dendritic structure with marked fluctuations in concentration between the dendritic regions (rich in Mn) and interdendritic ones (some excess of Fe and Co) in (**a**) dissappears after annealing in (**b**).
