# Supplementary figures and images for: Transition from High-Entropy to Conventional Alloys: Which Are Better?
Source: Materials (Basel). 2021 Oct 5;14(19):5824. doi: 10.3390/ma14195824 (PMC8510487; doi:10.3390/ma14195824)

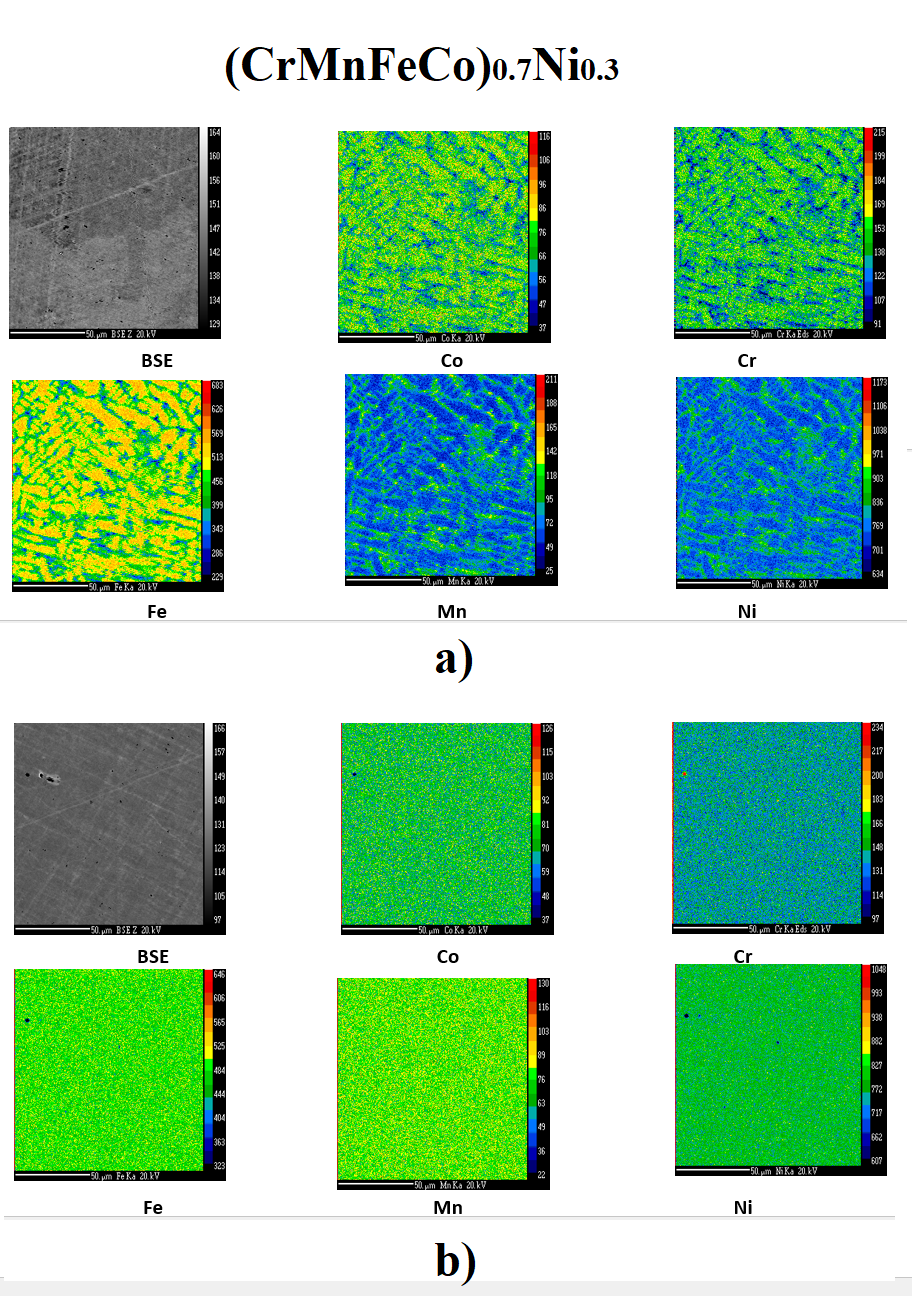

Supplement: Supplementary file 1 [file materials-14-05824-s001.zip › Figure_S1.tif]

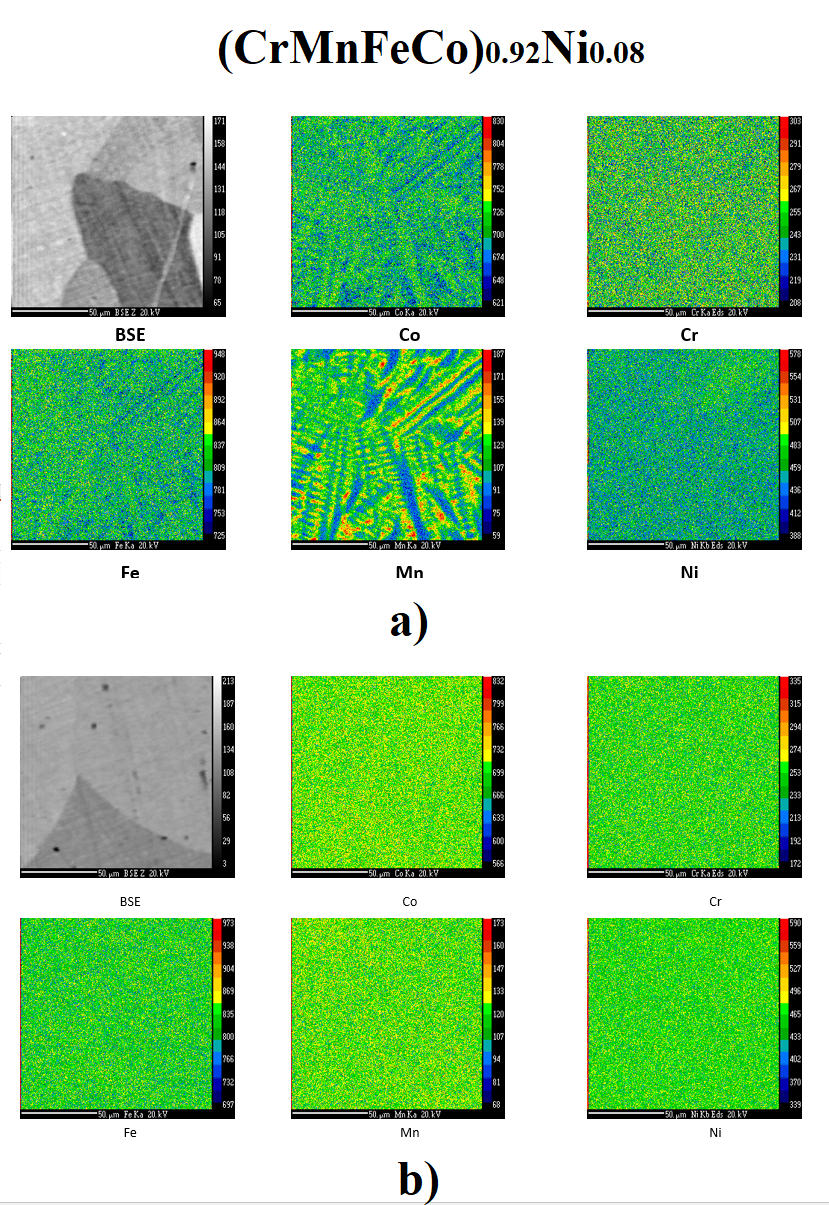

Supplement: Supplementary file 1 [file materials-14-05824-s001.zip › Figure_S2.tif]
